# Assessing the validity of a self-administered food-frequency questionnaire (FFQ) in the adult population of Newfoundland and Labrador, Canada

**DOI:** 10.1186/1475-2891-12-49

**Published:** 2013-04-16

**Authors:** Lin Liu, Peizhong Peter Wang, Barbara Roebothan, Ann Ryan, Christina Sandra Tucker, Jennifer Colbourne, Natasha Baker, Michelle Cotterchio, Yanqing Yi, Guang Sun

**Affiliations:** 1 Division of Community Health and Humanities, Faculty of Medicine, Memorial University, 300 Prince Philip Drive, St. John’s, NL, A1B 3V6, Canada; 2 School of Public Health, Tianjin Medical University, Tianjin, China; 3 First Affiliated Hospital, Wenzhou Medical College, Wenzhou, China; 4 Health Research Unit, Faculty of Medicine, Memorial University, St. John’s, Canada; 5 Population Study and Surveillance, Cancer Care Ontario, Toronto, ON, Canada; 6 Discipline of Medicine, Faculty of Medicine, Memorial University, St. John’s, Canada

**Keywords:** Food frequency questionnaire, Validity, Nutritional epidemiology

## Abstract

**Background:**

The Food- Frequency Questionnaire (FFQ) is a dietary assessment tool frequently used in large-scale nutritional epidemiology studies. The goal of the present study is to validate a self-administered version of the Hawaii FFQ modified for use in the general adult population of Newfoundland and Labrador (NL).

**Methods:**

Over a one year period, 195 randomly selected adults completed four 24-hour dietary recalls (24-HDRs) by telephone and one subsequent self-administered FFQ. Estimates of energy and nutrients derived from the 24-HDRs and FFQs were compared (protein, carbohydrate, fibre, fat, vitamin A, carotene, vitamin D, and calcium). Data were analyzed using the Pearson’s correlation coefficients, cross-classification method, and Bland–Altman plots.

**Results:**

The mean nutrient intake values of the 24-HDRs were lower than those of the FFQs, except for protein in men. Sex and energy-adjusted de-attenuated Pearson correlation coefficients for each nutrient varied from 0.13 to 0.61. Except for protein in men, all correlations were statistically significant with p < 0.05. Cross-classification analysis revealed that on average, 74% women and 78% men were classified in the same or adjacent quartile of nutrient intake when comparing data from the FFQ and 24-HDRs. Bland–Altman plots showed no serious systematic bias between the administration of the two instruments over the range of mean intakes.

**Conclusion:**

This 169-item FFQ developed specifically for the adult NL population had moderate relative validity and therefore can be used in studies to assess food consumption in the general adult population of NL. This tool can be used to classify individual energy and nutrient intakes into quartiles, which is useful in examining relationships between diet and chronic disease.

## Background

Food- Frequency Questionnaires (FFQs) are designed to assess habitual diet by asking about the frequency with which specific food items are consumed over a reference period [1,2]. This tool has been the most frequently used dietary assessment method in large-scale epidemiological studies and other nutritional research. Compared to other dietary assessment methods, the FFQ is easy to administer, has relatively low cost, and provides a rapid estimate of usual food intake [3]. However, investigators have recognized that nutritional values reported from FFQ data are subject to substantial error, both systematic and random, that can profoundly affect the design, analysis, and interpretation of nutritional epidemiologic studies [4,5]. For example, it is essential to covert food composition values from an FFQ into macronutrient and micronutrient values, but a major limitation in interpreting data from FFQs is the lack of homogeneity in food composition tables. Therefore, to properly interpret the results of epidemiological studies that use FFQs, it is necessary to know the relationship between reported intakes from the FFQ and true usual intakes [6]. Multiple dietary recalls [6-8], food records [9], and biomarkers [10] are generally considered to be more accurate reference measures of nutrient intake, and thus can be used in measuring the validity of FFQs. Validation correlations vary depending upon the nutrient, but typically range from 0.40 to 0.70 [8,11,12].

FFQs are widely used throughout the world for epidemiologic nutrition surveys. However, due to differences in food supply and dietary habits from one population to another, there is no universally accepted FFQ that can be used for all populations. A self-administrated FFQ, used for assessing the relationship between habitual diet and Colorectal Cancer (CRC) in adult residents of Newfoundland and Labrador (NL), was developed from the well-known Hawaii FFQ [13,14] and modified by NL researchers. Investigation of CRC in this population is warranted as NL has the highest CRC incidence rate in the country, when compared to other Canadian provinces [15]. The diets of residents of this province have been described as ‘unique’ due to the geography, economics, culture and population demographics [16], and thus an investigation into the possible relationship between dietary factors and CRC is especially warranted in NL. It has been suggested that elucidation of diet–disease relationships requires dietary assessment methods which can adequately describe and quantify intakes, minimize systematic errors and provide reasonably precise estimates of variability between individuals and/or groups [17]. However, the developed FFQ has not yet been appropriately validated for a NL population which makes some of the findings of the CRC study difficult to interpret.

Thus, the objectives of the present study are as follows: 1) to address whether this self-administered FFQ is valid in the NL general adult population by comparison with the results of multiple 24-hour dietary recalls (24-HDRs); and 2) to provide a validated NL based self-administrated FFQ for future use.

## Methods

### Sample recruitment and study design

Based on the information (means and standard deviations for various nutrients) derived from the FFQ data of the on-going CRC project [18-20] and the generally acceptable correlation coefficient value of 0.6, the minimum sample size for this study was determined to be 98 participants. The validation study lasted approximately one year and each subject was contacted a minimum of three times. A 30% attrition rate per step was expected. Therefore, an initial sample size of 450 subjects was required.

During February 2011, experienced telephone interviewers recruited a random population-based sample of NL adults, aged 35–70 years, using a list of land-line telephone numbers purchased from Info Canada [21]. After excluding non-residential telephone numbers, 683 potential subjects were identified as eligible and 432 (63%) initially agreed to participate in the study. Eligibility criteria included non-institutionalized adult resident of NL for at least two years with no intent to move in the next 12 months; aged 35–70 years inclusive at the time of the intended interviews; able to speak and read English at a grade 8 level; and with no specific identified medical conditions (cognitive impairment, psychological conditions, or pregnancy).

We collected dietary intake data by telephone through a set of two variably timed 24-HDRs (one weekday and one weekend day) from each participant, which then was duplicated approximately six months later. This procedure aimed to obtain two sets of recalls (a total of four 24-HDRs) in different seasons from each subject. An FFQ survey was mailed out to all study participants in early 2012, six months after the completion of the second pair of 24-HDRs. Reminder phone calls were used to prompt participants to complete and return the FFQs.

Demographic information, including: age, gender, size of their community, marital status, employment status, level of education, and smoking habits, was collected by telephone interview. This study was conducted according to the guidelines laid down in the Declaration of Helsinki and all procedures involving human subjects were approved by the Interdisciplinary Committee on Ethics in Human Research (ICEHR) [22], Memorial University (No. 2010/11-057-ME). Verbal informed consent was obtained from all subjects.

### Dietary assessment

#### The food-frequency questionnaire

The original Hawaii FFQ was designed to assess the typical food intake of individual males and females in a multi-ethnic Hawaiian/Southern Californian population [14]; it has been validated and widely used in the United States [23-25]. The FFQ administered in NL was modified to account for the unique food consumption habits in NL. Food items considered unusual in NL (e.g. tamales, ham hocks) were deleted or altered while some items commonly consumed in NL (e.g. moose meat, pickled meat) were added. This resulted in a list of 169 food and beverage items in the final instrument (available in Additional file 1).

The FFQ required participants to recall the number of times each food item was consumed per day, per week, per month, or rarely/never during the past 12 months. It also required participants to recall how many months of the year the food was consumed to account for seasonal variation in intake. Portion size options were given using standard measuring units (e.g. cups, tablespoons, slices) or by referring to photographs provided representing small, medium, and large portion sizes of some food items.

#### The 24-hour dietary recalls

The 24-HDRs were unannounced and conducted by telephone by trained interviewers. During the 24-HDR, each subject recalled and described in detail, all types and amounts of foods and beverages consumed in the previous 24 hours on two separate occasions, a weekday and a weekend day. Weekend days included Saturday and Sunday to capture food and alcohol consumption patterns which may be different from those on weekdays (Monday to Friday) [8,26,27]. The 24-hour period specified for the dietary recall was defined as the 24 consecutive hours between midnight on day one and midnight on the following day. To assist in estimating portion sizes of consumed foods, respondents were encouraged to view a measuring cup and measuring spoons as they completed their 24-HDR by telephone. At the end of this study, there were a total of four completed 24-HDRs for each participant.

### Statistical analysis

Data analyses attempted to (1) assess completeness of the responses and (2) examine potential errors/outliers. Both are directly related to overall validity assessment.

#### Data entry

Amounts and specific types/brands of foods consumed were entered into ESHA Food Processor SQL, version 10.8, nutrient analysis software (ESHA Research Inc, 2010, Salem, Oregon) [28] under the guidance of a professional Registered Dietitian and/or dietetic graduate students. This software contains more than 35,000 food and beverage items. When an exact match was not available between a food consumed and an item offered in the ESHA database, a group decision was made pertaining to the proper categorization of the food item in question. The group always included at least two dietetic professionals/students. For instance, homemade bread, which is not offered in the database, is known to contain more flour, honey or other ingredients than the commercial bread. Nutrient information from one slice of homemade bread was calculated as following:

$$\begin{array}{l} \;\mathrm{Nutrient}\;\mathrm{estimate}\;\mathrm{from}\;\mathrm{one}\;\mathrm{piece}\;\mathrm{of}\;\mathrm{homemade}\;\mathrm{bread}\\ =1.25\times \mathrm{Nutrient}\;\mathrm{estimate}\;\mathrm{from}\;\mathrm{one}\;\mathrm{piece}\;\mathrm{of}\\ \mathrm{commercial}\;\mathrm{bread} \end{array}$$

#### Calculation of nutrient intake

The nutrient composition of each item was obtained using the ESHA Food Processor. The nutrient composition data in the ESHA database is compiled from a variety of sources including the USDA Nutrient Database for Standard Reference, the USDA Database for the Continuing Survey of Food Intake by Individuals, the Canadian Nutrient File, manufacturers’ nutrient information, and over 1,000 additional sources of data.

Estimation of intake for a specific nutrient was conducted as following:

1. Within each round of 24-HDRs, each day was weighted appropriately to produce a synthetic week with the following formula:

$$\begin{array}{l} \mathrm{Mean}\;\mathrm{Daily}\;\mathrm{Nutrient}\;\mathrm{Estimate}\\ \;=(\left(\mathrm{Weekend}\;\mathrm{Intake}\times 2\right)\\ \;+\left(\mathrm{Weekday}\;\mathrm{Intake}\times 5\right))/7 \end{array}$$

2. Nutrient estimates from FFQ data were calculated using the product-sum method [1,29]. Thus,

$$\begin{array}{l} \mathrm{Daily}\;\mathrm{nutrient}\;\mathrm{intake}\\ =\sum [(\mathrm{reported}\;\mathrm{consumption}\;\mathrm{frequency}\;\mathrm{of}\\ \;a\;\mathrm{food}\;\mathrm{item},\mathrm{converted}\;\mathrm{to}\;\mathrm{times}\;\mathrm{per}\;\mathrm{day})\\ \;\times \left(\mathrm{portion}\;\mathrm{size}\;\mathrm{consumed}\;\mathrm{of}\;\mathrm{that}\;\mathrm{food}\right)\\ \;\times (\mathrm{amount}\;\mathrm{of}\;\mathrm{that}\;\mathrm{nutrient}\;\mathrm{in}\;a\;\mathrm{standard}\\ \;\mathrm{serving}\;\mathrm{size}\;\mathrm{of}\;\mathrm{that}\;\mathrm{food})] \end{array}$$

#### Validation study

Subjects were excluded if total energy intake from the FFQ fell outside the range of 500–5,000 kcal per day [1] (n = 4) or if more than one 24-HDR (n = 2) was rated as unreliable. We also excluded subjects with missing information (n = 4) from the analyses.

Means and standard deviations (SD) were calculated for nutrient intakes assessed by the 24-HDRs and FFQs. For the purpose of this study, the following nutrient intakes derived from the FFQ and 24-HDRs were compared: energy (kcal), protein, total fat, saturated fat, monounsaturated fat, polyunsaturated fat, carbohydrate, dietary fibre, cholesterol, carotene, calcium, vitamin A, and vitamin D. Paired-sample t-tests were used to determine differences between the means for energy and nutrients derived from the two dietary tools. All nutrient variables were log-transformed to improve normality and reduce skewness, and then were energy-adjusted using the residual method [30].

The relationship between the nutrient values from the FFQ, both the unadjusted and the energy-adjusted nutrient estimates, and averages of the two synthetic weeks of recalls were estimated using Pearson correlation coefficients. We also calculated de-attenuated correlations to remove the within-person variability found in the recalls [31] by using the following formula:

$$r_{t}\;=\;r_{0}\sqrt{1\;+\;r/n}$$

Here **r**_**t**_ is the corrected correlation between the energy-adjusted nutrient derived from the FFQ and 24-HDRs, **r**_**0**_ is the observed correlation, **r** is the ratio of the within- and between-person variance measured from the 24HDRs, and **n** is the number of replicated recalls (n = 4).

Furthermore, we categorized the distribution of energy-adjusted nutrient intakes into quartiles, and estimated the percentage of subjects classified into same, adjacent and extreme quartiles [10,32,33]. The Bland–Altman method [34] was also used to assess the agreement between the mean energy and nutrient intake values obtained using the two different instruments. We plotted the difference in intake between the two methods (FFQ-24HDR) against the mean intake of the two measures ((FFQ + 24HDR)/2). The overall mean difference indicated whether one method tends to overestimate or underestimate, and the limits of agreements (mean ±1.96 SD) were used to show how well the two administrations agree.

All analyses were conducted using the SAS statistical software package version 9.2 (SAS Institute Inc., Cary, NC, USA) and Statistical Package for Social Science (SPSS) software version 9.0 (SPSS, Inc., Chicago, IL, USA).

## Results

**Figure 1:**
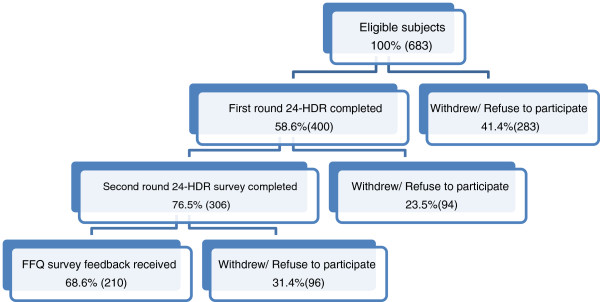
Flow diagram of sample selection.

Out of the 432 participants who agreed to participate in this study, 400 (93%) completed the first two dietary recalls; of these, 306 (77%) completed the second round of 24-HDRs and 210 (49%) completed the FFQ (Figure 1). After excluding those with unreliable data (n = 15), 195 subjects (153 females, 42 males) were included in the present analysis. The mean (SD) age of the 195 participants was 55.03 (8.75) years. Over half of the participants were employed (53.3%), were rural residents (56.9%), and the majority had completed post-secondary education (60.5%), were non-smokers (82.6%), and were married (78.5%). When comparing the demographic characteristics of the participants at baseline and the 1-year follow-up visit, no significant differences were observed (data not shown).

Table 1 presents the means and respective standard deviations for energy and nutrients, derived from the FFQ and 24-HDRs. Values for energy and nutrients estimated by the FFQ were higher than those obtained using the dietary recalls, except for protein in men. Evaluation of the differences between these means showed significant differences (p < 0.05) for all the nutrients in women and some nutrient estimates in men (dietary fibre, vitamin A, vitamin D, and calcium).

**Table 1:** Comparison of nutrient intakes per day by Food Frequency Questionnaire (FFQ) and 24-Hour Recall (24-HDR)

	**Women**			**Men**		
	**FFQ**	**24-HDRs**^**a**^	***p-value***	**FFQ**	**24-HDRs**^**a**^	***p-value***
**Energy (kcal)**	2130.93(751.47)^b^	1505.33(496.50)	0.00*	2138.52(737.47)	2001.68(604.74)	0.26
**Protein (g)**	86.83(30.81)	63.58(19.81)	0.00*	89.01(36.35)	89.95(26.13)	0.89
**Carbohydrate (g)**	265.12(106.35)	188.88(66.55)	0.00*	256.27(100.97)	240.80(85.38)	0.24
**Dietary Fibre (g)**	22.56(11.68)	14.33(5.93)	0.00*	20.12(10.39)	16.49(6.14)	0.02*
**Total Fat (g)**	83.62(35.79)	55.42(23.84)	0.00*	80.87(31.71)	73.74(26.98)	0.24
**Saturated Fat (g)**	26.75(12.25)	16.91(8.15)	0.00*	26.48(11.85)	22.90(9.05)	0.10
**Monounsaturated Fat (g)**	30.52(14.39)	17.91(8.76)	0.00*	28.34(11.90)	25.65(11.30)	0.27
**Polyunsaturated Fat (g)**	15.26(7.64)	10.09(4.86)	0.00*	14.59(6.20)	12.89(5.06)	0.15
**Cholesterol (mg)**	288.00(193.69)	214.44(104.62)	0.00*	299.12(155.72)	282.91(105.23)	0.56
**Vitamin A(RAE)**	1133.14(622.12)	490.21(260.20)	0.00*	1050.41(897.80)	623.66(357.54)	0.01*
**Carotene (RE)**	624.33(699.23)	338.61(354.25)	0.00*	499.81(272.40)	416.53(417.71)	0.27
**Vitamin D (IU)**	275.42(162.57)	137.39(79.26)	0.00*	287.69(178.28)	192.32(100.24)	0.00*
**Calcium (mg)**	1073.17(561.17)	561.37(240.67)	0.00*	1043.57(615.55)	710.97(328.90)	0.00*

^a^ Average of two round of weighted 24-HDRs.

^b^ Values are given as Mean (Standard Deviation).

* Significance of the difference between mean 24-HDR and FFQ estimates (p-value < 0.05).

Correlations between nutrient intakes derived from the FFQs and the 24-HDRs are shown in Table 2 for men and women. The Pearson correlation coefficient for crude data varied from 0.17 (carbohydrate) to 0.40 (carotene) in women and 0.07(protein) to 0.56 (carbohydrate) in men. In both genders, adjusting for total energy intake improved the correlations in some nutrients (e.g. protein) but decreased the values in the others (e.g. polyunsaturated fat). However, adjustment for residual measurement error (de-attenuation) increased all correlations, ranging from 0.20 (polyunsaturated fat) to 0.52 (dietary fibre) in women and 0.13 (protein) to 0.61 (carbohydrate, dietary fibre) in men, with a median correlation value of 0.38 in women and 0.42 in men. Except for that of protein in men, all correlations were statistically significant with p < 0.05.

**Table 2:** Pearson correlations between Food Frequency Questionnaire (FFQ) estimates and weighted 24-Hour Recall (24-HDR) estimates

**Nutrient^a^**	**Women**			**Men**		
	**Unadjusted**	**Adjusted**^**b**^	**De-attenuated**	**Unadjusted**	**Adjusted**^**b**^	**De-attenuated**
**Energy (kcal)**	0.23	—	0.26*	0.39	—	0.44*
**Protein (g)**	0.25	0.30	0.36*	0.07	0.11	0.13
**Carbohydrate (g)**	0.17	0.34	0.38*	0.56	0.54	0.61*
**Dietary Fibre (g)**	0.32	0.47	0.52*	0.55	0.54	0.61*
**Total Fat (g)**	0.33	0.32	0.37*	0.24	0.32	0.38*
**Saturated Fat (g)**	0.27	0.28	0.33*	0.28	0.26	0.31*
**Monounsaturated Fat (g)**	0.36	0.29	0.34*	0.23	0.41	0.51*
**Polyunsaturated Fat (g)**	0.29	0.17	0.20*	0.23	0.20	0.26*
**Cholesterol (mg)**	0.25	0.34	0.44*	0.10	0.33	0.42*
**Vitamin A(RAE)**	0.26	0.32	0.38*	0.23	0.35	0.42*
**Carotene (RE)**	0.40	0.38	0.50*	0.13	0.19	0.28*
**Vitamin D (IU)**	0.32	0.37	0.45*	0.41	0.45	0.55*
**Calcium (mg)**	0.32	0.43	0.50*	0.50	0.45	0.51*

^a^ All nutrients were log-transformed to improve normality.

^b^ Nutrient intakes were adjusted for total energy intake by residual method.

*Correlations of 0.16 and higher have a p-value less than 0.05.

Data for energy-adjusted nutrient intakes estimated from the FFQs and 24-HDRs were distributed into quartiles of intakes and cross-classified. A subject would be correctly classified if his/her energy or nutrient intakes were ranked into the same or an adjacent quartile by both methods. Table 3 presents the summary of cross-classification analysis. For women, classification of subjects into the same and adjacent quartiles ranged from 66.7% (polyunsaturated fat) to 79.1% (dietary fibre), while grossly misclassified individuals varied from 3.3% (carbohydrate, dietary fibre) to 9.1% (polyunsaturated fat). For men, the mean proportion of individuals correctly classified was 78.0%, while on average only 5.85% fell into the extreme quartile. Bland–Altman plots showed no serious systematic bias between the administration of the two instruments over the range of mean intakes (plots were shown in the Additional file 2).

**Table 3:** Percentage for cross-classification of energy-adjusted nutrient intakes into quartiles estimated from the Food Frequency Questionnaire (FFQ) and 24-Hour Recalls (24-HDRs)

**Nutrient^a^**	**Women**				**Men**			
	**Same quartile (%)**	**Adjacent quartile (%)**	**One quartile apart (%)**	**Grossly misclassified (%)**	**Same quartile (%)**	**Adjacent quartile (%)**	**One quartile apart (%)**	**Grossly misclassified (%)**
**Energy (kcal)**	24.8	43.1	24.2	7.8	40.5	33.3	21.4	4.8
**Protein (g)**	33.3	41.2	18.3	7.2	23.8	45.2	23.8	7.1
**Carbohydrate (g)**	32.7	45.1	19.0	3.3	40.5	45.2	12.0	2.3
**Dietary Fibre (g)**	40.5	38.6	17.6	3.3	38.1	42.9	19.0	0.0
**Total Fat (g)**	34.6	35.3	23.5	6.5	31.0	42.9	16.6	9.5
**Saturated Fat (g)**	32.7	42.5	17.6	7.2	28.6	50.0	14.3	7.1
**Monounsaturated Fat (g)**	37.9	33.3	20.3	8.5	38.1	45.2	9.5	7.1
**Polyunsaturated Fat (g)**	34.0	32.7	24.2	9.1	33.3	42.9	14.3	9.5
**Cholesterol (mg)**	32.7	36.6	24.2	6.5	40.5	40.5	11.9	7.1
**Vitamin A(RAE)**	30.1	42.5	20.3	7.2	26.2	47.6	21.4	4.8
**Carotene (RE)**	37.9	39.2	16.3	6.5	31.0	40.5	21.4	7.1
**Vitamin D (IU)**	38.6	39.2	18.3	3.9	23.8	57.1	14.3	4.8
**Calcium (mg)**	31.4	45.8	19.0	3.9	33.3	52.4	9.5	4.8

^a^ Classification was performed using log-transformed nutrient values.

## Discussion

A valid, comprehensive tool to measure nutrient intakes is essential to health research involving humans, especially when it is aimed at investigating the relationship between diet and diseases [35,36]. The present study demonstrated that a previously developed 169-item self-administered FFQ is reasonably valid for dietary assessment in the general adult population of NL. We observed high agreement between the two methods investigated in quartile categorization, as more than 74% women and 78% men were correctly classified into the same or adjacent quartiles for energy and twelve nutrients. Bland-Altman plots also indicated acceptable level of agreement between the two methods.

A major component of the validation process is the selection of an appropriate reference method to test the target instrument; however no gold standard exists for dietary intake measurements. It is crucial for the errors of both the methods used in the current study to be as independent of each other as possible [37]. In a review on the validation of FFQs, Cade *et al. (2002)* found that 75% of the studies validated FFQs against repeated 24-HDRs [3]. The FFQ and the 24-HDRs have some similar error sources, such as the reliance on memory and the perception of portion sizes [1,3]; however, the FFQ stresses long-term memory whereas the 24-HDR relies on short-term memory. In addition, the 24-HDR method was interviewer-based using open-ended questions, whereas the FFQ was self-administered with close-ended questions. Such differences let us assume that the errors are sufficiently independent and that the 24-HDR method is an adequate comparison method for this target instrument [38].

The present study sample was comparable to the general population with regard to geographical distribution [39]. There were significant more females than males who participated in the study. The reason may be that females are willing to care about nutrition intake and health than males [40]. Sakamaki *et al. (2005)* also found that females had significantly greater desire to be on a weight-loss diet than males (p < 0.001). In addition, individuals with a higher education level and those who were non-smokers were more likely to participate in the study. These may lead to potential sources of bias, which must be kept in mind when interpreting the results.

As expected, the absolute nutrient values derived from the FFQ tended to be higher than those derived from the 24-HDRs, which is a common issue reported in previous research [17,29,35,41]. A possible explanation is that people tend to overestimate their actual intake when they are asked to recall the frequency of a large number of foods consumed in an FFQ [1,29]. According to nutrient intakes of NL adults estimated in 2004 by the Canadian Community Health Survey (CCHS Cycle 2.2) [42], all nutrient intakes estimated by the current study were within the acceptable range (±20%) of the mean values.

Correlation coefficients were used to assess the association between FFQ and 24-HDRs as well as to measure the relative validity. For both genders, energy adjustment improved the correlations for the majority of nutrients. According to Willett [30], energy adjustment increases correlation coefficients when the variability of nutrient consumption is related to energy intake, but decreases correlation coefficients when the variability depends on systematic errors of overestimation and underestimation. In the present study, the lower correlation values found in some categories may indicate that the FFQ to some extent systemically over-/under- estimated intake of these nutrients, however, error in over/under estimation by the FFQ is expected. Likewise, Dehghan *et al. (2012)*, Wang *et al. (2008)*, and Cardoso *et al. (2010)* found energy adjustment did not improve the crude correlation in their studies [43-45].

Due to correction for the day-to-day variation in intakes, the de-attenuated energy-adjusted correlations were usually higher than their original values. On average, the correlation values were approximately 0.40 when genders were combined. These values are lower than some reported by previous validation studies [6,10,14] but comparable to others [8,11,46-48]. In regards to energy, lower concordance coefficients have been reported in the Willett FFQ (0.16 for women and 0.18 for men) and the Block FFQ (0.37 for women and 0.41 for men) [11] as compared with 0.26 (women) and 0.44 (men) derived from our study. It was particularly noticeable that our correlations for protein were unfavourable, especially in men (0.13), however, our findings were similar to those obtained from a Brazilian cohort (0.20) [45]. For carbohydrate in women, our study yielded a coefficient of 0.38, which compares favourably with the Jackson Heart Study (0.32) [46]. Our low correlations for polyunsaturated fat (0.20 for women and 0.26 for men) were very similar to the results of most other FFQs [11,46-48]. This could be associated in part with xthe irregular distribution of oils used in food preparation. In terms of micronutrients, it has been suggested that the number of days which must be monitored to allow a true estimation of average daily intake is greater for micronutrients than for macronutrients and exceeds the four days investigated in this study [49]. Although our correlations for vitamin A in women (0.38) and carotene in men (0.28) were low, they were significant with *p-value* <0.05, suggesting reasonably good agreement between the two instruments. Other studies have also reported poor correlations for micronutrients [6,44,45], including vitamin A and carotene.

The use of correlation analysis for assessing validity has often been questioned on the basis that it does not measure agreement but only measures the strength of association between two variables [50,51]. Cross-classification into quartiles of intake and Bland–Altman plots were therefore used to achieve a measure of the agreement between the two methods. In terms of total energy and all nutrients analyzed, this FFQ shows a relatively high proportion of subjects being correctly classified (into same or adjacent category) and only a small number of grossly misclassified individuals (less than 10%). As a result, we demonstrated stronger between-method agreement than other studies [52,53]. This may reflect a high sensitivity for this instrument. Bland–Altman plots showed no systematic bias for most of the nutrients evaluated by the FFQ and the dispersion between the mean intakes estimated by the two instruments suggests a good concordance trend for some nutrients, such as dietary fibre.

Several limitations of this study must be considered. First, we did not administer an FFQ at the onset of the study, thus cannot assess the reproducibility of the instrument. Future work needs to be done to evaluate the reproducibility (reliability) of this FFQ. Furthermore, relevant information pertaining to the use of dietary supplements was not collected during the 24-HDRs. Therefore, we do not know the true nutrient intakes of this population. As well, majority participants in the present study were females, non-smokers or with higher education levels. These may result in over-or under-represented individuals in the specific groups, also may create potential sources of bias. Finally, as in most research, the general limitations of dietary assessment instruments cannot be ignored. Both the FFQ and 24-HDR methods rely on memory and may be biased due to under- or over-estimation. It has been suggested by others that multiple reference methods, including dietary methods and biochemical analyses, be used in validation studies [3,36] to increase the accuracy of the results. Future studies may benefit from including biomarker reference methods such as urinary nitrogen and doubly labeled water; however, using a biomarker will certainly add to the participant burden and costs associated with the study. As well, it is noteworthy that use of the FFQ remains the most cost-effective way to collect usual nutrient intakes in population studies.

## Conclusion

In conclusion, this 169-item FFQ developed specifically for the NL population had moderate relative validity and therefore can be used in studies to assess food consumption in the NL general population. In addition, this FFQ is capable of classifying an individual’s intake into quartiles, which is useful in examining the relationships between diet and chronic disease including CRC. Such a validation is not only immediately assisting the analyses and interpretation of data collected during the CRC study, but also contributes greatly to future epidemiological studies and other nutritional studies in NL. Further efforts should be made to evaluate the reproducibility of the present FFQ.

## Competing interests

The authors declare that they have no competing interests.

## Authors’ contributions

This work was derived from LL’s MSc thesis. PPW and BR were committee members and contributed both the conceptualization and the fruition of this work. LL conducted the literature search, data analysis, and finalized the manuscript. AR, YY, MC, GS, CT, CJ, BN, the co-project investigators, contributed to the execution of this study at various stages. All authors read and approved the final manuscript.

## Supplementary Material

Additional file 1 Food Frequency Questionnaire. Click here for file

Additional file 2 Bland–Altman plots. Click here for file
