# Supplementary material for: Assessing the validity of a self-administered food-frequency questionnaire (FFQ) in the adult population of Newfoundland and Labrador, Canada
Source: Nutr J. 2013 Apr 16;12:49. doi: 10.1186/1475-2891-12-49 (PMC3637508; doi:10.1186/1475-2891-12-49)
Supplement: Additional file 1 — Food Frequency Questionnaire. [file 1475-2891-12-49-S1.pdf]

Canadian Study of Diet and Health

Memorial University of Newfoundland

Who this questionnaire is for and what it asks about:

This questionnaire is to be completed by the person taking part in this study:

Part I asks about the foods you ate **OVER THE PAST 12 MONTHS**.

Part II asks about vitamins and other dietary supplements that you may have used.

If possible, please return this questionnaire within two weeks.

The completed questionnaire should be sealed in the pre-paid envelope and mailed back to:

Health Research Unit,
Room 2801A, Division of Community Health & Humanities,
Medicine, Health Science Centre,
300 Prince Phillip Drive,
St. John's, NL, Canada, A1B 3V6

If you have any questions about this form or the study, please do not hesitate to contact with us with **709-777-2043**; leave your question when the answering machine indicates and someone will get back to you within the day.

The information given to us in this questionnaire will be kept confidential.

Thank you for your time and assistance.

HOW TO COMPLETE THIS QUESTIONNAIRE

We would like to know how often you ate certain foods **OVER THE PAST 12 MONTHS,** and their amounts.

Section A ***(lists foods and portion sizes)***
Amounts are described in various ways, including the number of:

cups, teaspoons (tsp), ounces (oz), inches ("), pieces (e.g., 1 apple)
grams (gm), tablespoons (tbsp), millilitres (ml), centimetres (cm).

We want to know the ***Portion Size*** of your **USUAL SERVING.** We have given an example of an average portion size in the attached pamphlet. If your portion size was different than the average, you can indicate this by putting an **X** or ✓ in the circles for ***Smaller*** or ***Larger*** portion sizes. ***Smaller*** than average is about 25% or less than the average portion size, while ***Larger*** than average is about 25% or more than the average size. Leave the circle blank if your typical portion size was average.

One part of the attached pamphlet shows small, medium and large portion sizes for vegetables, meat and chicken. Some questions ask you to refer to the photos to help you figure out correct ***Food Portion Size.***

Section B ***(asks about how often you ate certain foods OVER THE PAST 12 MONTHS)***
For each food item listed, choose **one** column (Per Day, Per Week, Per Month, or Never / Rarely) that best describes ***HOW OFTEN*** you ate or drank that item. For example, if you ate CREAM CHEESE 3 times a month during the year of interest, you would write (3) in the *PER MONTH* column. If you ate SWEET POTATOES only 2 times during the year of interest, you can place a checkmark (✓) in *the NEVER OR RARELY* column.

Section C ***(To be completed only for seasonal foods)***
Some foods (for example fresh fruit and vegetables) are not available throughout the year. **For foods that you do not eat all year round** (i.e. in season only), indicate the number of months of the year that you ate them.

Please complete each question as best you can. We know that it is difficult to recall exactly how often you ate something. If you are not certain, try to give your best estimate. If you need help completing the FFQ please call (**709)-777-2043**. If the call is outside the St. John's area you can call collect.

EXAMPLE

	Section A				Section B OVER THE PAST 12 MONTHS				Section C
	**FOOD**	**Average Portion Size**	**Your Portion Size, if NOT Average** Smaller	Larger	**HOW OFTEN?** (Complete one column only) per DAY (enter a number)	per WEEK (enter a number)	per MONTH (enter a number)	NEVER or RARELY (check)	**If Ate Food in Season Only** enter Months per Year
1	CREAM CHEESE	2 tbsp/ 30 ml/ 1 oz	O	O			3		
2	CANTELOUPE	1/8 or 1 slice	O	O		1			4
3	SWEET POTATOES	1 medium/ ½ cup	O	O				√	

Section A				Section B<br>OVER THE PAST 12 MONTHS				Section C
	FOOD	Average Portion Size	Your Portion Size, if NOT Average<br>Smaller Larger	HOW OFTEN? (Complete one column only)<br>per **DAY** (enter a number)	per **WEEK** (enter a number)	per **MONTH** (enter a number)	**NEVER** or RARELY (check)	**If Ate Food In Season Only**<br>enter Months per Year
	**Beverages**							
1	WHOLE MILK (any, include if in cereal & drinks)	1 cup/ 250 ml	O O					
2	2% MILK, 2% Evaporated milk (any, if in cereal & drinks)	1 cup/ 250 ml	O O					
3	SKIM MILK, 1% MILK (any, include if in cereal & drinks)	1 cup/ 250 ml	O O					
4	MILK SHAKE	1 cup/ 250 ml	O O					
5	YOGURT DRINK	1 cup/ 250 ml	O O					
6	COFFEE (not decaffeinated)	1 cup/ 250 ml	O O					
7	COFFEE (decaffeinated)	1 cup/ 250 ml	O O					
8	TEA (not herbal)	1 cup/ 250 ml	O O					
9	TEA (herbal)	1 cup/ 250 ml	O O					
10	SUGAR (in tea and coffee)	1tsp or 1 cube	O O					
11	COCA COLA, PEPSI, OTHER COLA	1 cup/ 250 ml	O O					
12	DIET SOFT DRINKS	1 cup/ 250 ml	O O					
13	OTHER SOFT DRINKS (not dietetic or cola)	1 cup/ 250 ml	O O					
14	ORANGE or GRAPEFRUIT JUICE	¾ cup/ 175 ml	O O					
15	APPLE or GRAPE JUICE	¾ cup/ 175 ml	O O					
16	OTHER FRUIT JUCES (pineapple, cranberry, etc)	¾ cup/ 175 ml	O O					
17	FRUIT DRINK/ LEMONADE	¾ cup/ 175 ml	O O					
18	FRUIT DRINKS, ICED TEA	¾ cup/ 175 ml	O O					
19	VEGTABLE JUICES	¾ cup/ 175 ml	O O					
20	BEER or ALE	335 ml/ 1 bottle	O O					
21	WHITE WINE	150 ml / 5 oz	O O					
22	RED WINE, SHERRY, PORT (or other fortified wine)	150 ml / 5 oz	O O					

Section A				Section B OVER THE PAST 12 MONTHS				Section C
FOOD		Average Portion Size	Your Portion Size, if NOT Average	HOW OFTEN? (Complete one column only)				If Ate Food In Season Only
			Smaller Larger	per DAY (enter a number)	per WEEK (enter a number)	per MONTH (enter a number)	NEVER or RARELY (check)	enter Months per Year
23	LIQUOR (for example, whiskey, rum etc)	45 ml/ 1.5 oz	O O					
	**Dairy Products**							
24	EGG (boiled, poached)	1 medium	O O					
25	EGG (fried, scrambled, omelette)	1 medium	O O					
26	CREAM CHEESE, Regular fat	2 tbsp/ 30 ml/ 1 oz	O O					
27	CHEESE, Regular fat (such as cheddar, Swiss, processed)	1 slice/ 30 g/ 1oz	O O					
28	CHEESE, Light (6-15% fat, such as cream cheese, cheddar)	1 slice/ 30 g/ 1oz	O O					
29	CHEESE, Ultra Light (5% fat or less, such as cheddar)	1 slice/ 30 g/ 1oz	O O					
30	COTTAGE or RICOTTA CHEESE	125 ml/ ½ cup	O O					
31	CREAM (coffee, whipping, sour or regular)	1 tbsp/ 15 ml	O O					
32	CREAM (half and half, light sour cream)	1 tbsp/ 15 ml	O O					
33	COFFEE WHITENER (non-dairy)	1 tbsp/ 15 ml	O O					
34	YOGURT, Regular (plain, 2.4% fat or more)	6 oz/ 170 g	O O					
35	YOGURT, Light (plain, less than 2.4% fat)	6 oz/ 170 g	O O					
36	YOGURT, Regular (fruit flavoured or frozen, 2.4% fat or more)	6 oz/ 170 g	O O					
37	YOGURT, Light (fruit flavoured or frozen, less than 2.4% fat)	6 oz/ 170 g	O O					
	**Mixed Dishes**							
38	SOUPS (creamed)	1 cup/ 250 ml	O O					
39	SOUPS (non-creamed)	1 cup/ 250 ml	O O					
40	PEA SOUP	1 cup/ 250 ml	O O					
41	PASTA with meat sauce (spaghetti, lasagna)	1 cup/ 250 ml	O O					
42	PASTA with tomato sauce (spaghetti)	1 cup/ 250 ml	O O					
43	MIXED DISHES with cheese or cheese sauce (macaroni and cheese)	1 cup/ 250 ml	O O					
44	PIZZA with meat	1 medium slice	O O					

Section A									Section C
					Section B OVER THE PAST 12 MONTHS				
	FOOD	Average Portion Size	Your Portion Size, if NOT Average		HOW OFTEN? (Complete one column only)				If Ate Food In Season Only
			Smaller	Larger	per **DAY** (enter a number)	per **WEEK** (enter a number)	per **MONTH** (enter a number)	**NEVER** or RARELY (check)	enter Months per Year
45	PIZZA with vegetable only	1 medium slice	O	O					
46	MEAT STEW with carrots, other vegetables	1 cup/ 250 ml/ photo A, medium	O	O					
47	CHILI with meat or Con Carne	1 cup/ 250 ml	O	O					
	**Vegetables**								
48	POTATOES (mashed, boiled, baked etc)	1 medium/ ½ cup/ 125 ml	O	O					
49	FRENCH FRIES or FRIED POTATOES	1 cup/ 250 ml	O	O					
50	CARROTS (raw or cooked)	1 medium/ ½ cup /125 ml	O	O					
51	BROCCOLI	1 cup/ 250 ml	O	O					
52	CABBAGE, COLESLAW	½ cup/ 125 ml	O	O					
53	CAULIFLOWER	½ cup/125 ml	O	O					
54	CORN	1 ear / ½ cup	O	O					
55	PEAS or LIMA BEANS	½ cup/125 ml	O	O					
56	GREEN or YELLOW BEANS	½ cup/125 ml	O	O					
57	BEANS or LENTILS (baked or boiled beans, kidney beans, chickpeas)	½ cup/125 ml cooked	O	O					
58	SPINACH and other green leafy vegetables (greens, collards, kale, mustard greens etc)	½ cup cooked or 1 cup raw	O	O					
59	GREEN SALAD (with lettuce)	1 cup/ 250 ml	O	O					
60	CUCUMBER	½ cup/ 125 ml sliced	O	O					
61	TOMATOES (fresh)	1 medium/ ½ cup/ 125 ml	O	O					
62	TOMATOES (canned, pureed or sauce)	½ cup/125 ml	O	O					
63	ONIONS (raw or cooked)	½ cup/125 ml	O	O					
64	BEETS (boiled or pickled)	½ cup/125 ml	O	O					
65	TURNIPS or RUTABAGAS	1 medium/ ½ cup/125 ml	O	O					
66	OTHER ROOT VEGETABLES (sweet potatoes, yams, radish, etc)	½ cup/125 ml	O	O					

Section A					Section B OVER THE PAST 12 MONTHS				Section C
	FOOD	Average Portion Size	Your Portion Size, if NOT Average Smaller	Larger	HOW OFTEN? (Complete one column only) per DAY (enter a number)	per WEEK (enter a number)	per MONTH (enter a number)	NEVER or RARELY (check)	If Ate Food In Season Only enter Months per Year
67	YELLOW SQUASH (winter type)	½ cup/125 ml	O	O					
68	ZUCCHINI or EGGPLANT	½ cup/125 ml	O	O					
69	SWEET PEPPER (green, red or yellow)	½ cup/125 ml	O	O					
70	ASPARAGUS or BRUSSEL SPROUTS	½ cup/125 ml	O	O					
71	BEAN SPROUTS, ALFALFA SPROUTS	½ cup/125 ml	O	O					
72	PICKLES, RELISH	1 dill/ 2 tbsp	O	O					
73	AVOCADO	½ medium	O	O					
74	OTHER VEGETABLES (celery, mushrooms, etc., raw or cooked)	½ cup/125 ml	O	O					
	**Meats and Fish**								
75	GROUND BEEF, Regular (hamburger, meat loaf, in casseroles etc)	85 g/ 3 oz/ 3" patty	O 2 oz	O 4 oz					
76	GROUND BEEF, Medium (hamburger, meat loaf, in casseroles etc)	85 g/ 3 oz/ 3" patty	O 2 oz	O 4 oz					
77	GROUND BEEF, Lean (hamburger, meat loaf, in casseroles etc)	85 g/ 3 oz/ 3" patty	O 2 oz	O 4 oz					
78	ROAST BEEF	photo B, medium	O	O					
79	STEAK	photo B, medium	O	O					
80	PORK CHOP	photo B, medium	O	O					
81	ROAST PORK	photo B, medium	O	O					
82	BAKED HAM	photo B, medium	O	O					
83	BACON	2 slices	O	O					
84	VEAL	photo B, medium	O	O					
85	LAMB	photo B, medium	O	O					
86	HOT DOG or WIENER (Enter buns/rolls under item 116)	1 hot dog/ 2 oz	O	O					
87	SAUSAGE	85 g/ 3 oz	O	O					
88	CORNED BEEF	1 slice	O	O					

Section A					*Section B* OVER THE PAST 12 MONTHS				*Section C*
	**FOOD**	**Average Portion Size**	**Your Portion Size, if NOT Average**		**HOW OFTEN?** (Complete one column only)				**If Ate Food In Season Only** enter Months per Year
			Smaller	Larger	per **DAY** (enter a number)	per **WEEK** (enter a number)	per **MONTH** (enter a number)	**NEVER** or RARELY (check)	
89	COLDCUTS (ham, salami, bologna, etc)	1 medium slice/ 30g/ 1 oz	O	O					
90	LIVER	85 g/ 3 oz	O	O					
91	FRIED CHICKEN	photo C, medium	O	O					
92	CHICKEN / TURKEY (roasted or stewed)	photo C, medium	O	O					
93	CHICKEN / TURKEY, SKIN REMOVED	photo C, medium	O	O					
94	SALTED/ DRIED MEAT	photo C, small	O	O					
95	PICKLED MEAT (brined)	photo C, small	O	O					
96	SHELLFISH (shrimp, lobster, crab)	85 g/ 3 oz/ photo C, small	O	O					
97	FRIED FISH	170 g/ 6 oz/ photo B, medium	O	O					
98	FISH (baked or broiled)	170 g/ 6 oz/ photo B, medium	O	O					
99	CANNED FISH (tuna, salmon)	½ can/ 48 ml/ 1.7 oz	O	O					
100	SMOKED FISH or LOX	85 g/ 3 oz/ photo C, small	O	O					
101	SALTED/ DRIED FISH	85 g/ 3 oz/ photo C, small	O	O					
102	PICKLED FISH	85 g/ 3 oz/ photo C, small	O	O					
103	SEA-BIRDS, SEAL	85 g/ 3 oz/ photo C, small	O	O					
104	CARIBOU, MOOSE	85 g/ 3 oz/ photo C, small	O	O					
105	PARTRIDGE, OTHER WILD BIRDS	85 g/ 3 oz/ photo C, small	O	O					
	**Cereals and Grains**								
106	BRAN or GRANOLA CEREALS (including All Bran)	½ cup/ 125 ml	O	O					
107	WHOLE WHEAT CEREALS (such as shredded wheat)	½ cup/ 125 ml/ 1 biscuit	O	O					
108	CEREALS, NOT SUGAR COATED (such as Special K)	½ cup/ 125 ml	O	O					
109	HOT CEREALS (for example: oatmeal)	½ cup/ 125 ml	O	O					
110	SUGAR COATED CEREALS	½ cup/ 125 ml	O	O					
111	OTHER BREAKFAST CEREALS	½ cup/ 125 ml	O	O					

Section A				Section B<br>OVER THE PAST 12 MONTHS				Section C
FOOD	Average Portion Size	Your Portion Size, if NOT Average<br>Smaller	Larger	HOW OFTEN? (Complete one column only)<br>per **DAY** (enter a number)	per **WEEK** (enter a number)	per **MONTH** (enter a number)	**NEVER** or RARELY (check)	**If Ate Food In Season Only**<br>enter Months per Year
112 SUGAR ON CEREAL	1 tsp	O	O					
113 100% WHOLE GRAIN or DARK BREAD	1 slice	O	O					
114 60% WHOLE GRAIN, LIGHT RYE	1 slice	O	O					
115 WHITE BREAD	1 slice	O	O					
116 WHITE BREAD ROLLS (including hot dog buns etc)	1 roll	O	O					
117 WHOLE WHEAT ROLLS	1 roll	O	O					
118 CRACKERS (snack or soda type)	5	O	O					
119 BRAN/OAT MUFFIN	1 medium, ½ extra large	O	O					
120 OTHER MUFFIN (plain cake, with berries)	1 medium, ½ extra large	O	O					
121 PANCAKES, WAFFLES	1 medium	O	O					
122 MACARONI, SPAGHETTI, NOODLES (plain)	1 cup cooked/ 250 ml	O	O					
123 RICE	½ cup cooked/ 125 ml	O	O					
124 CRISP SNACKS (potato chips, popcorn, pretzels etc)	1 cup	O	O					
**Fruits**								
125 APPLE, PEAR	1 medium	O	O					
126 CITRUS FRUITS (orange, grapefruit)	1 orange, ½ grapefruit	O	O					
127 BERRIES (strawberries, blueberries, bakeapples)	½ cup/ 125 ml	O	O					
128 GRAPES	½ cup/ 125 ml	O	O					
129 BANANA	1 medium	O	O					
130 PEACH, PLUM, NECTARINE, APRICOT	1 medium	O	O					
131 CANTALOUPE	1/8 or 1 slice	O	O					
132 WATERMELON	1 wedge, 3" base	O	O					
133 HONEYDEW MELON	1/8 or 1 slice	O	O					
134 MANGO	1 medium	O	O					
135 PAPAYA	1 medium	O	O					

	FOOD	Average Portion Size	Your Portion Size, if NOT Average: Smaller	Larger	per DAY (enter a number)	per WEEK (enter a number)	per MONTH (enter a number)	NEVER or RARELY (check)	If Ate Food In Season Only: enter Months per Year
	Section A				*Section B* OVER THE PAST 12 MONTHS — HOW OFTEN? (Complete one column only)				*Section C*
136	APPLESAUCE	½ cup/ 125 ml	O	O					
137	DRIED FRUITS (raisins, dates, prunes)	2 tbsp/ 2 dates	O	O					
138	CANNED FRUIT (all kinds)	½ cup/ 125 ml	O	O					
139	ALL OTHER FRUIT (fresh kiwi, pomegranate, etc.)	1 medium	O	O					
	**Desserts and Sweets**								
140	CAKES	1 slice, 2" x 4" x 1"	O	O					
141	PIES and TARTS	1 slice	O	O					
142	DONUTS and SWEET ROLLS	1	O	O					
143	COOKIES	1	O	O					
144	ICE CREAM	½ cup/ 125 ml	O	O					
145	LIGHT or DIET ICE CREAM	½ cup/ 125 ml	O	O					
146	PUDDING	½ cup/ 125 ml	O	O					
147	DIET or LIGHT PUDDING	½ cup/ 125 ml	O	O					
148	JELLO	½ cup/ 125 ml	O	O					
149	POPSICLES, FREEZIES	1	O	O					
150	CHOCOLATE BAR and CHOCOLATE CANDY	1 bar / 50g or 5 candy size	O	O					
151	CANDY (without chocolate)	1 caramel	O	O					
	**Miscellaneous**								
152	TOFU, TEMPEH	½ cup, 2" x 2" x 1" piece	O	O					
153	KETCHUP	1 tbsp	O	O					
154	MAYONNAISE/ MIRACLE WHIP, Regular fat (on bread, salad, meat, etc)	1 tbsp	O	O					
155	MAYONNAISE/ MIRACLE WHIP, Light (on bread, salad, meat, etc)	1 tbsp	O	O					
156	SALAD DRESSING, Regular fat (French, Italian etc)	1 tbsp	O	O					
157	OIL (in cooking)	1 tbsp	O	O					

Section A				Section B OVER THE PAST 12 MONTHS				Section C
FOOD	Average Portion Size	Your Portion Size, if NOT Average Smaller	Larger	HOW OFTEN? (Complete one column only) per **DAY** (enter a number)	per **WEEK** (enter a number)	per **MONTH** (enter a number)	**NEVER** or RARELY (check)	**If Ate Food In Season Only** enter Months per Year
158 BUTTER (on vegetables or bread; exclude use in baked and mixed dishes)	1 pat/ 1 tbsp	O	O					
159 MARGARINE (on vegetables or bread; exclude use in baked or mixed dishes)	1 pat/ 1 tbsp	O	O					
160 PEANUT BUTTER	1 tbsp	O	O					
161 PEANUTS	30g/ 1 oz	O	O					
162 OTHER NUTS	30g /1 oz	O	O					
163 JAM, JELLY, HONEY, SYRUP	1 tbsp	O	O					
164 GRAVY	4 tbsp	O	O					
165 CHOCOLATE or STRAWBERRY SYRUP	1 tbsp	O	O					
166 CHOCOLATE SPREADS	1 tbsp	O	O					
167 SAUCES (white, cream, Mornay)	30 ml/ 1oz/ 2 tbsp	O	O					
168 WHEAT BRAN	1 tbsp	O	O					
169 WHEAT GERM	1 tbsp	O	O					

Continue on next page →

PART 2 - USE OF VITAMINS AND DIETARY SUPPLEMENTS

Now we would like to know about your use of vitamins and dietary supplements.
OVER THE PAST 12 MONTHS, did you take any of the following? If *Yes*, then specify usual brand and amount and how long you took them.

Vitamin and Amount – if used, ⇒	How many pills did you take per week?	How long had you taken them?
**Vitamin C** EXAMPLE<br>O None O Below 500 ☒ 500-1000 O above 1000 mg	0 5 per week	2 4 months
**Multivitamins that include minerals** ⇒<br>O No O Yes If yes, usual brand ____________	per week	months
**Multivitamins, no minerals**<br>O No O Yes If yes, usual brand ____________	per week	months
**B Complex vitamins**<br>O No O Yes If yes, usual brand ____________	per week	months
**In the following items, DO NOT INCLUDE use of the above MULTIVITAMINS**		
**Vitamin A**<br>O None O Below 10000 O 10000-15000 O above 15000 IU	per week	months
**Vitamin C**<br>O None O Below 500 O 500-1000 O above 1000 mg	per week	months
**Vitamin E**<br>O None O Below 400 O 400-800 O above 800 IU	per week	months
**Beta-carotene**<br>O None O Below 10000 O 10000-15000 O above 15000 IU	per week	months
**Folic acid**<br>O None O Below 1.0 O 1.0 mg O above 1.0 mg*	per week	months
**Calcium**<br>O None O Below 250 O 250-500 O above 500 mg	per week	months
**Iron**<br>O None O Below 100 O 100-200 O above 200 mg	per week	months
**Other dietary supplements (e.g., yeast, cod liver oil, etc)**<br>O No O Yes, specify type: ____________	per week	months
____________	per week	months

* 1 mg = 1000 micrograms

Thank you very much for completing this questionnaire! Because we want to be able to use all the information you have provided, we would greatly appreciate it if you would please take a moment to review each page making sure that you:

- Did not skip any page
- Completely erased any changes you may have made

We welcome any other information or comments that you would like to give us:

____

____

____

THANK YOU VERY MUCH for your assistance in this research!

For Office Use Only

Study #: ____________________

Interviewer: ____________________

Date completed (D/M/Y): ______________________
