# Supplementary material for: Assessing the validity of a self-administered food-frequency questionnaire (FFQ) in the adult population of Newfoundland and Labrador, Canada
Source: Nutr J. 2013 Apr 16;12:49. doi: 10.1186/1475-2891-12-49 (PMC3637508; doi:10.1186/1475-2891-12-49)

Additional Files for Bland–Altman plots:

Plot for Total Energy intake (female)

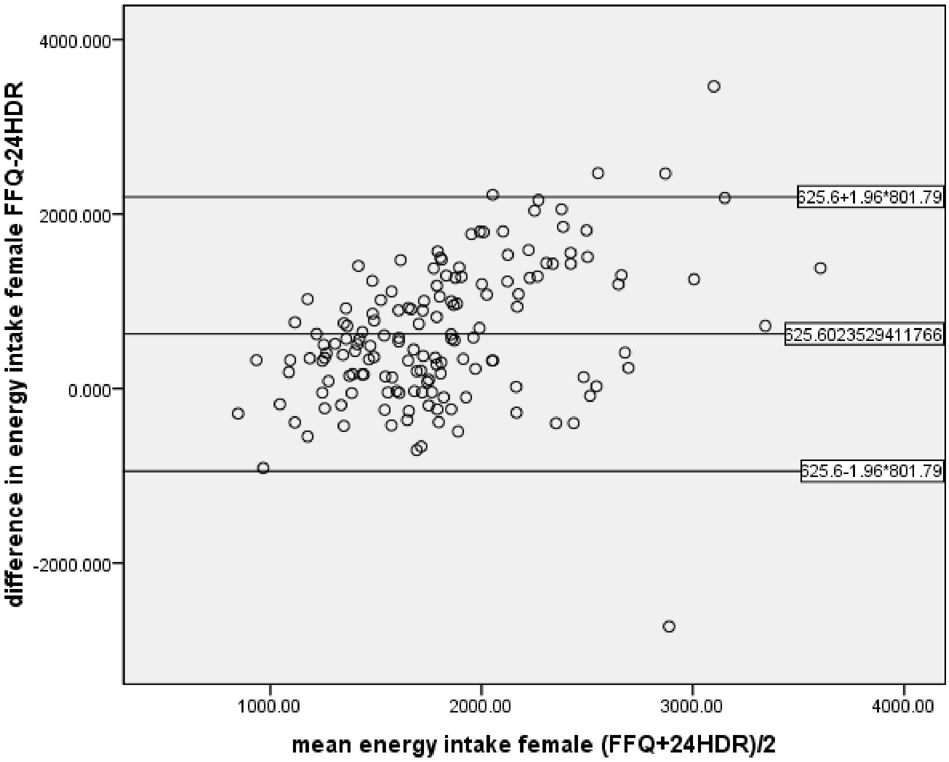

Plot for Protein intake (female)

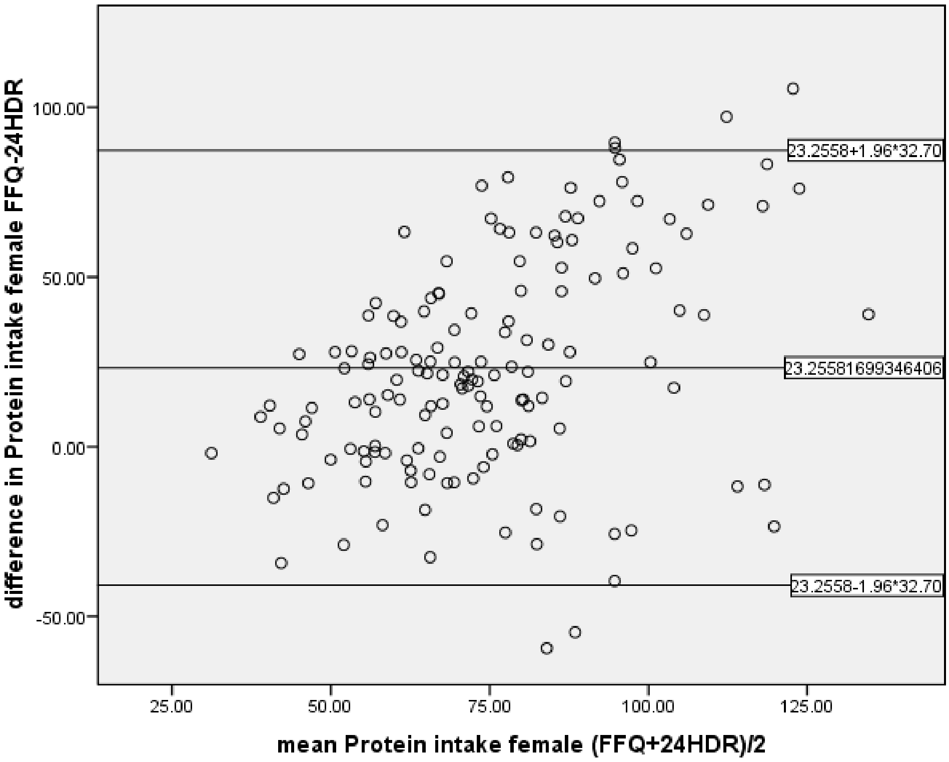

Plot for Carbohydrate intake (female)

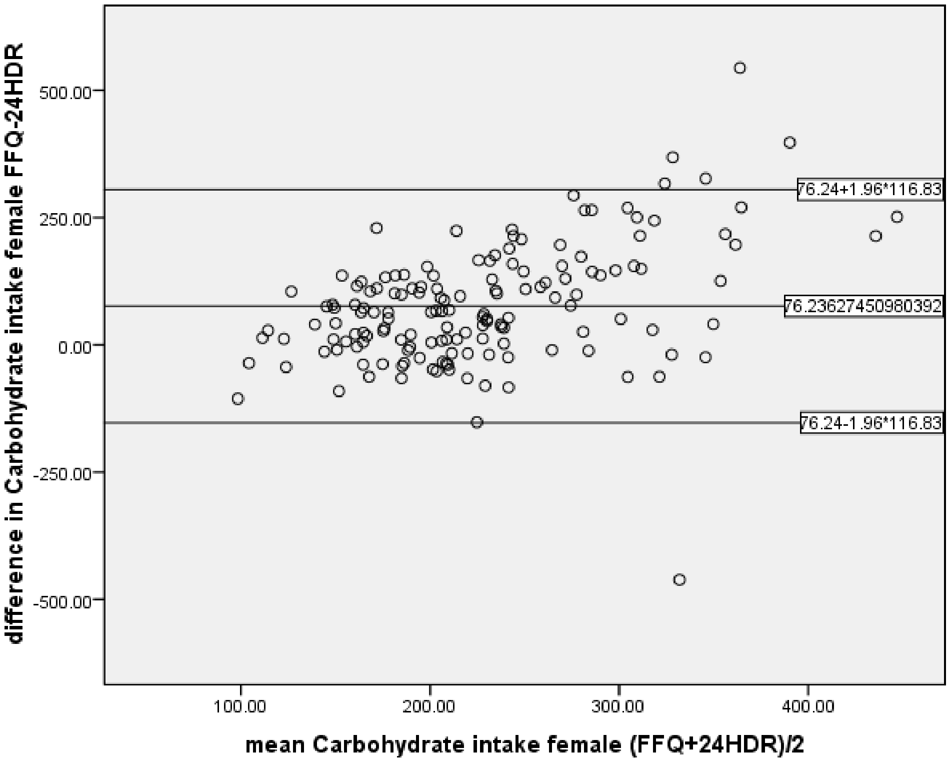

Plot for Dietary Fibre intake (female)

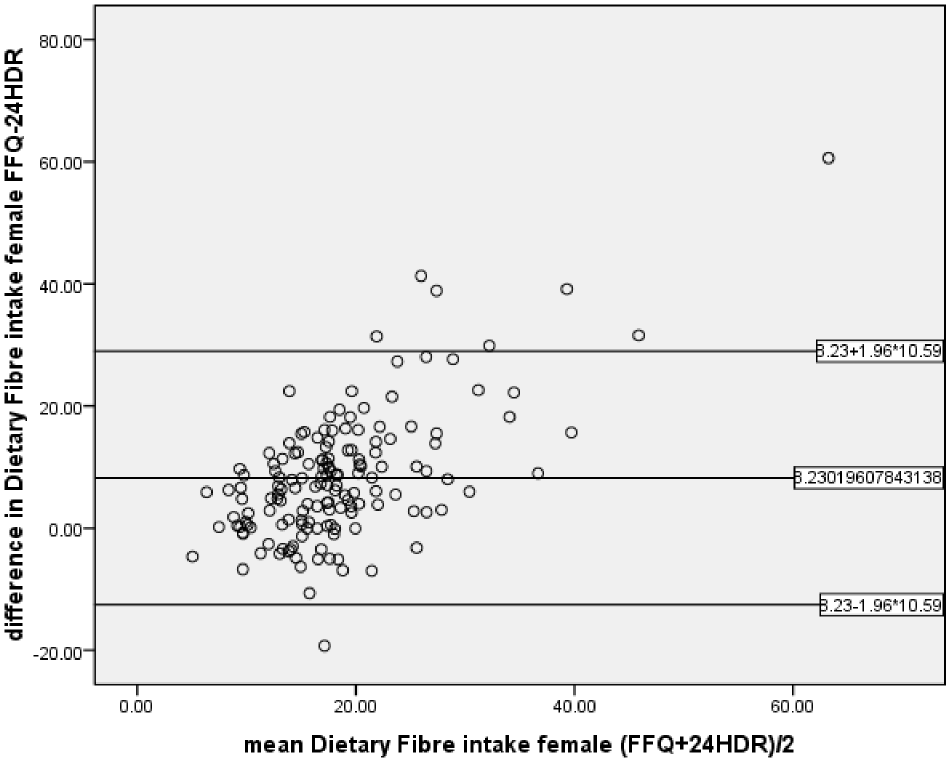

Plot for Total Fat intake (female)

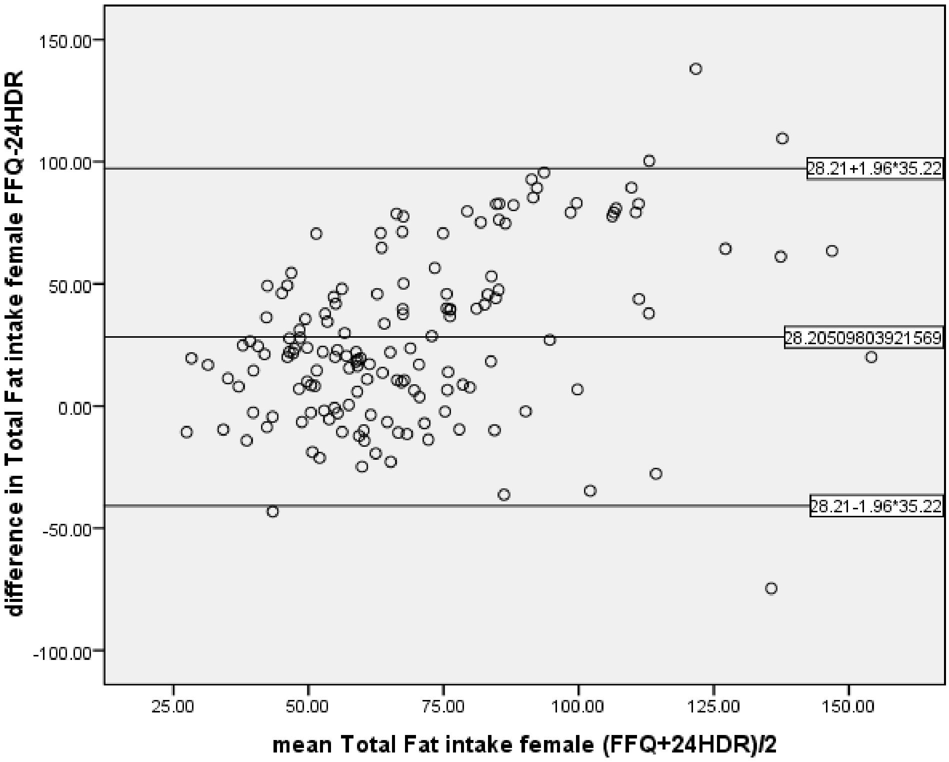

Plot for Saturated Fat intake (female)

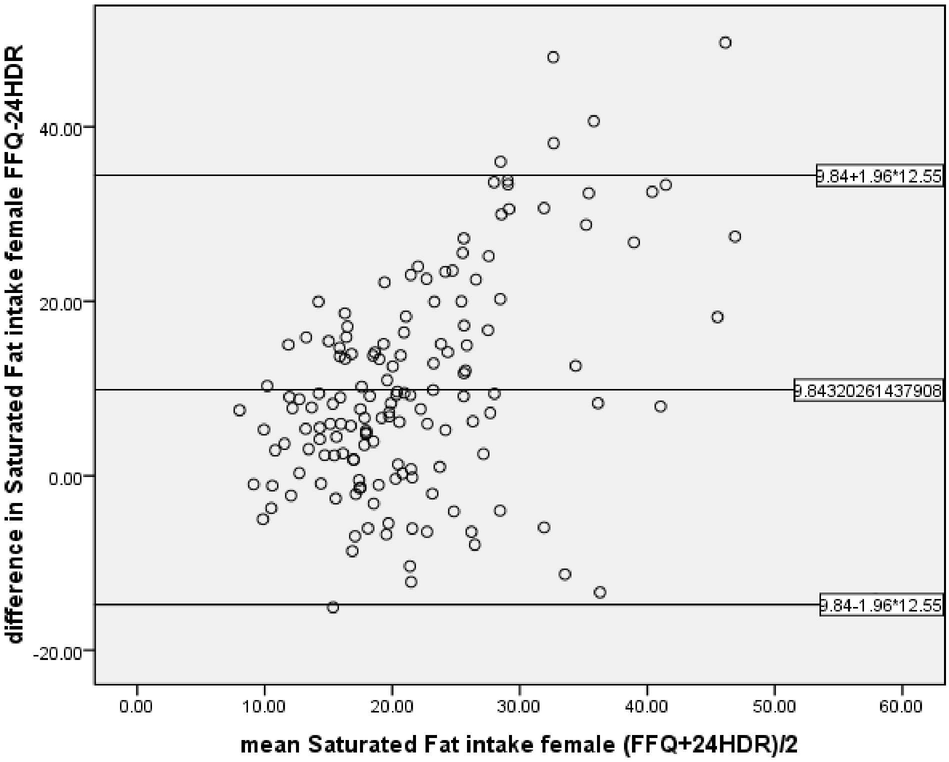

Plot for Monounsaturated Fat intake (female)

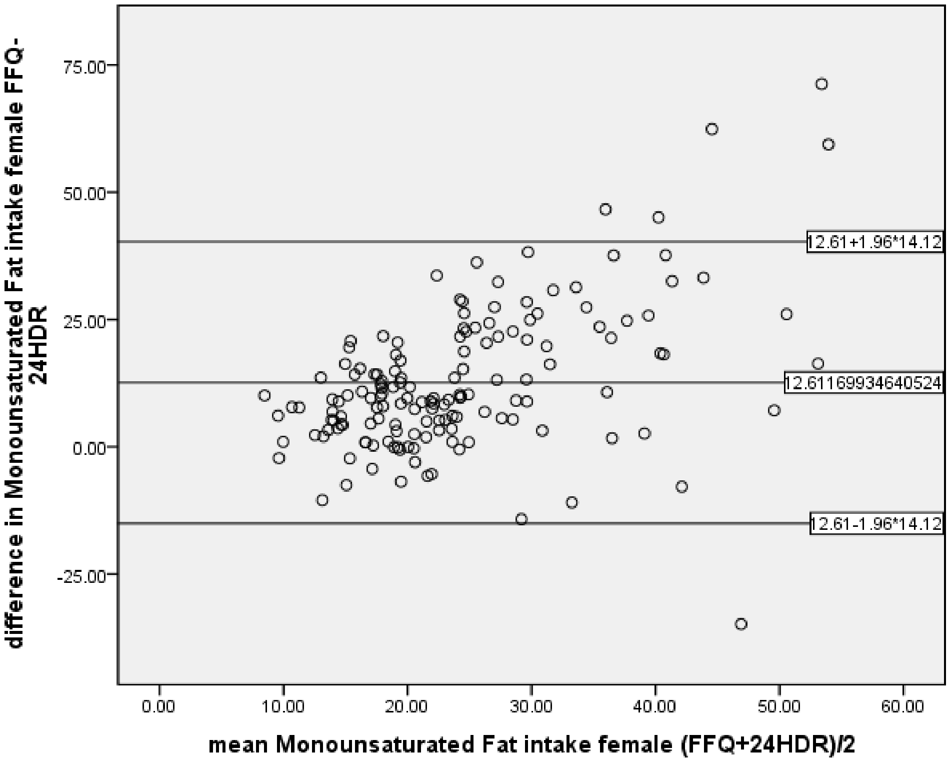

Plot for Polyunsaturated Fat intake (female)

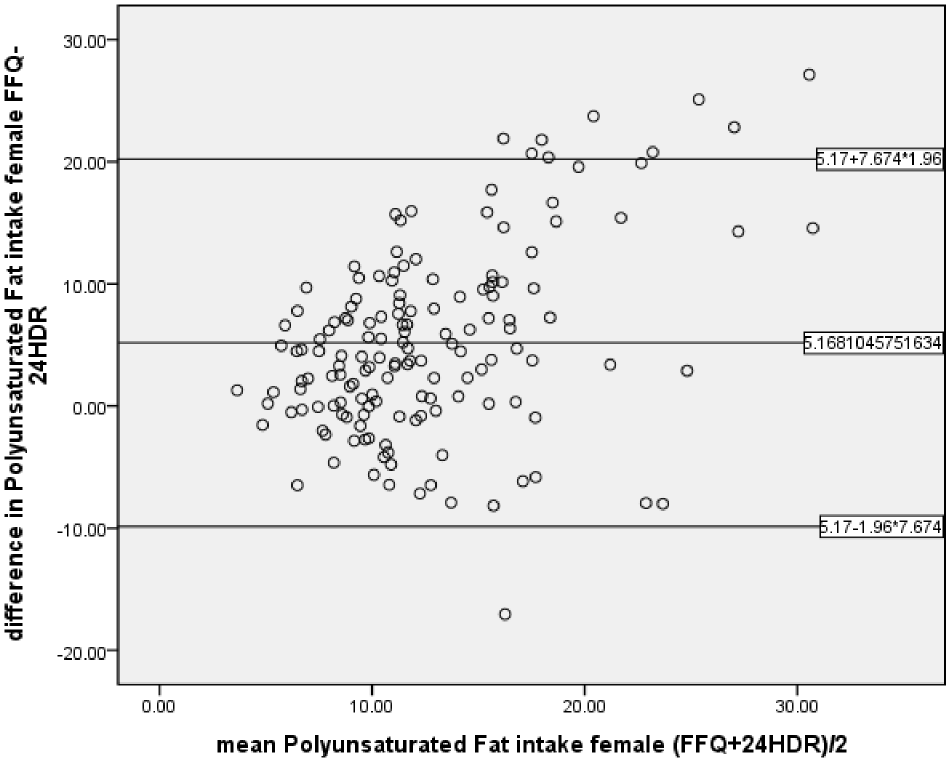

Plot for Cholesterol intake (female)

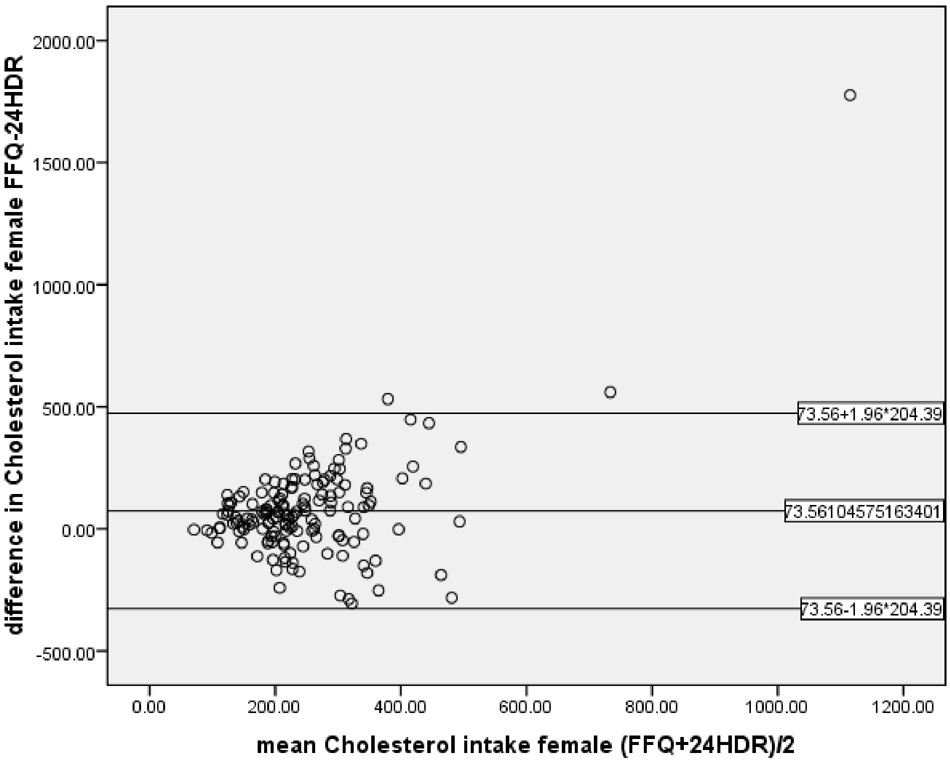

Plot for Vitamin A intake (female)

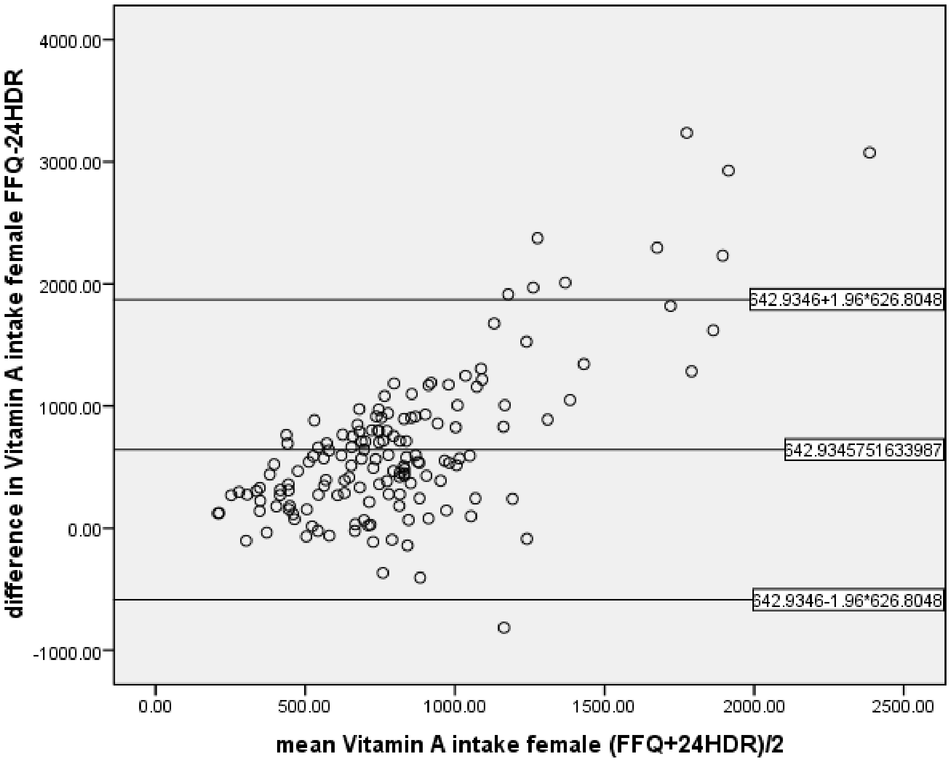

Plot for Carotene intake (female)

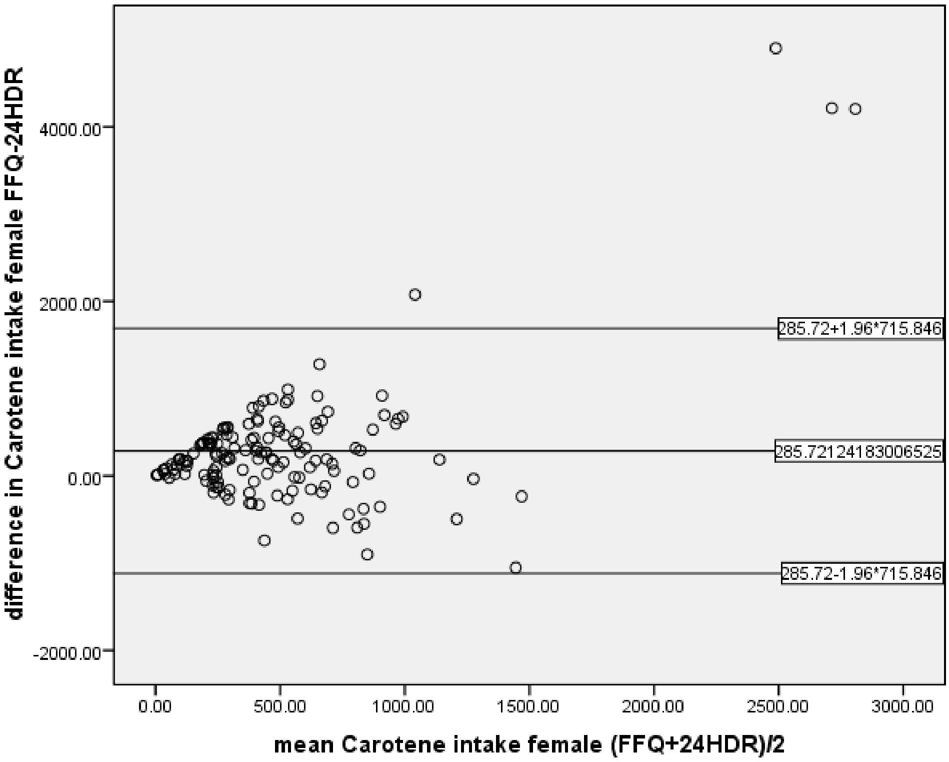

Plot for Vitamin D intake (female)

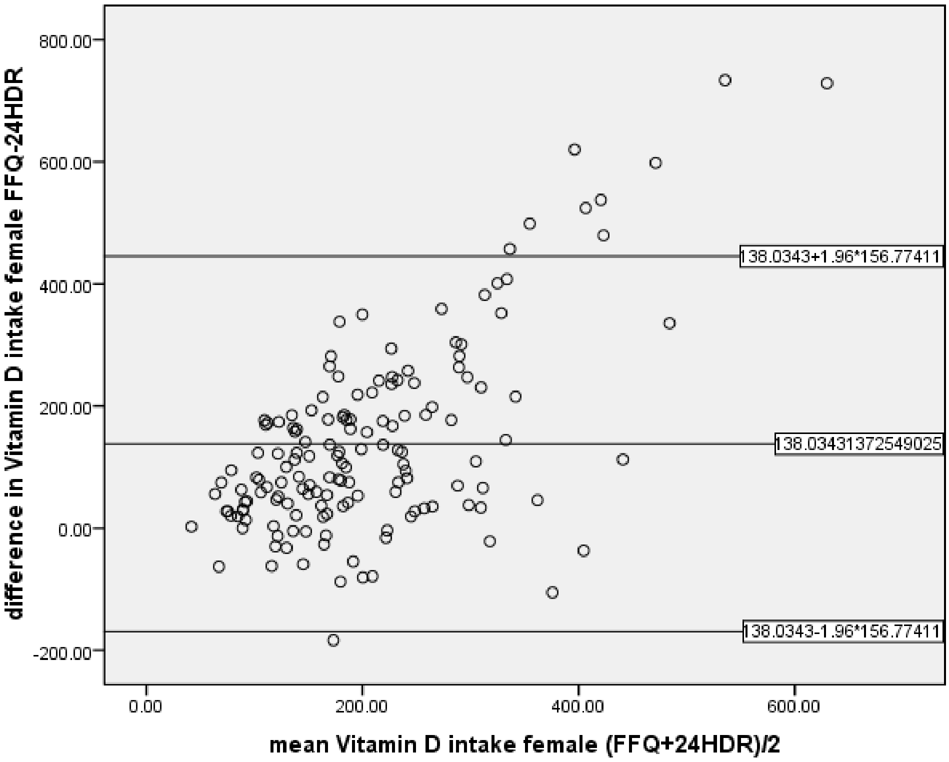

Plot for Calcium intake (female)

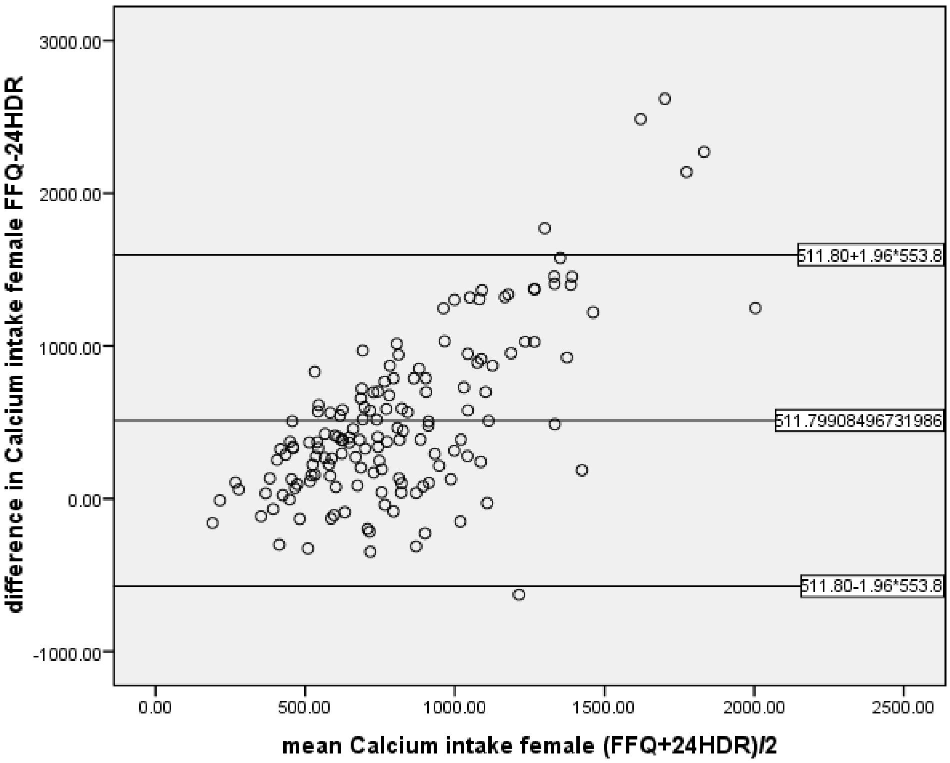

Plot for Total Energy intake (male)

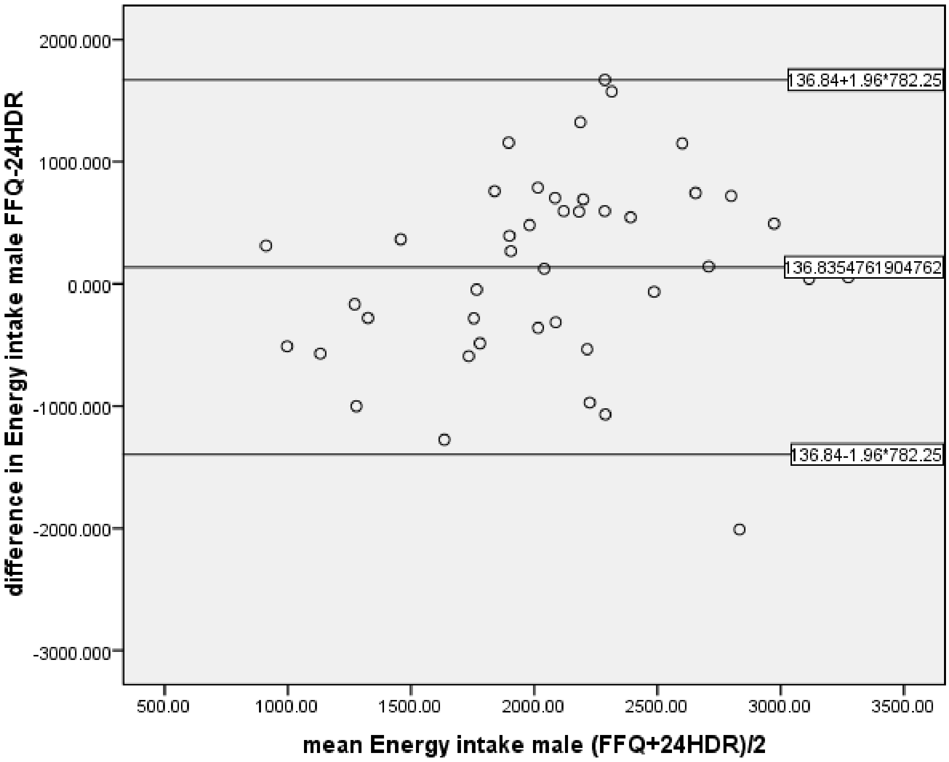

Plot for Protein intake (male)

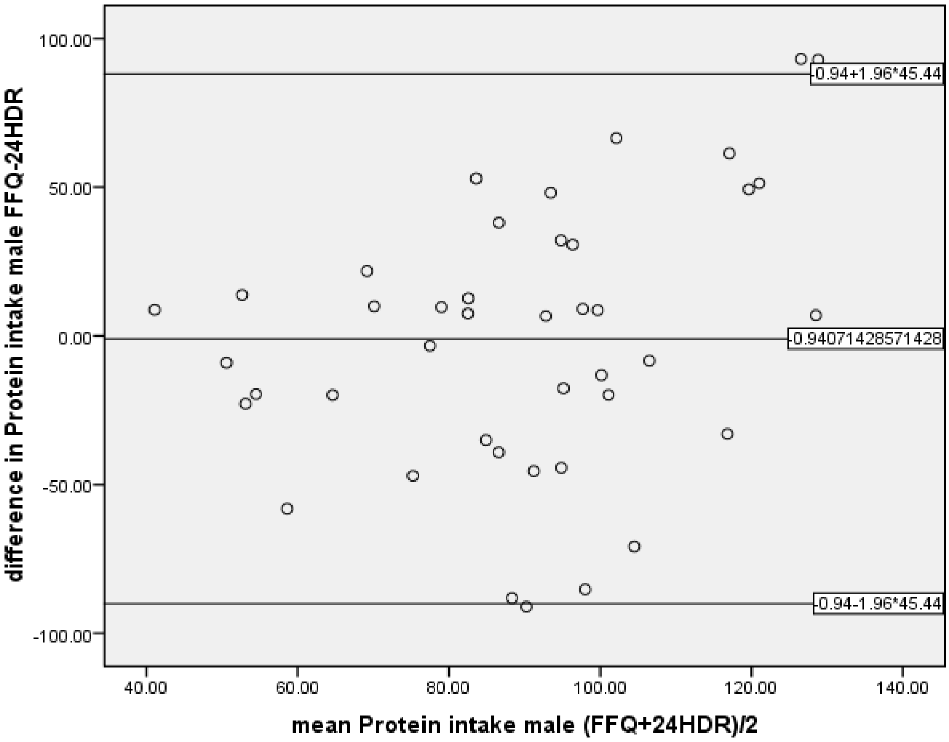

Plot for Carbohydrate intake (male)

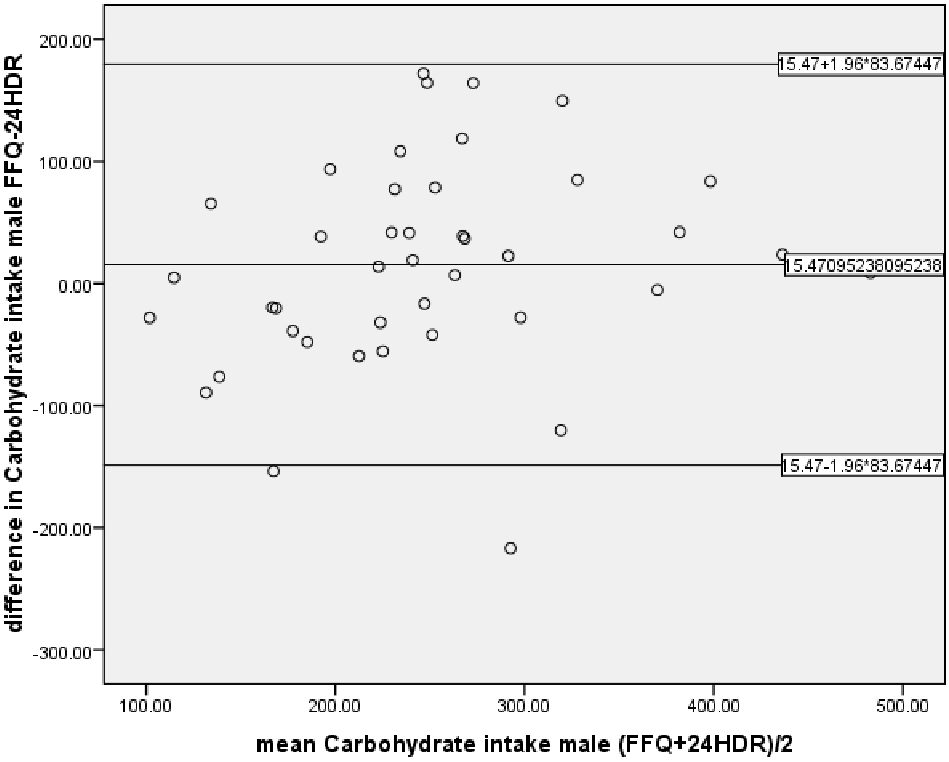

Plot for Dietary Fibre intake (male)

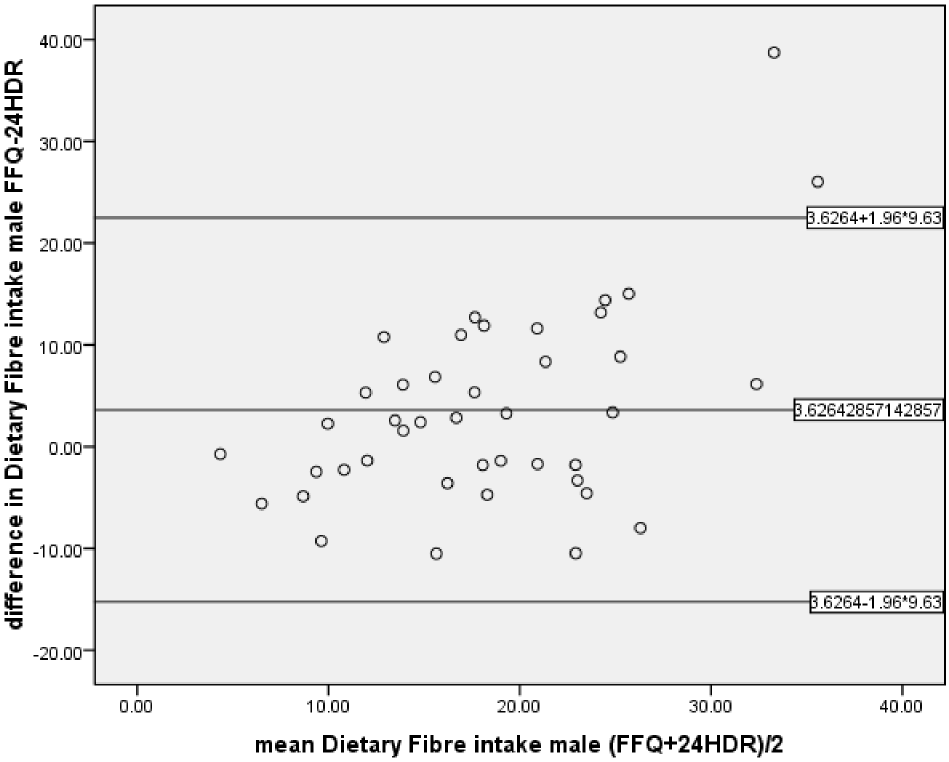

Plot for Total Fat intake (male)

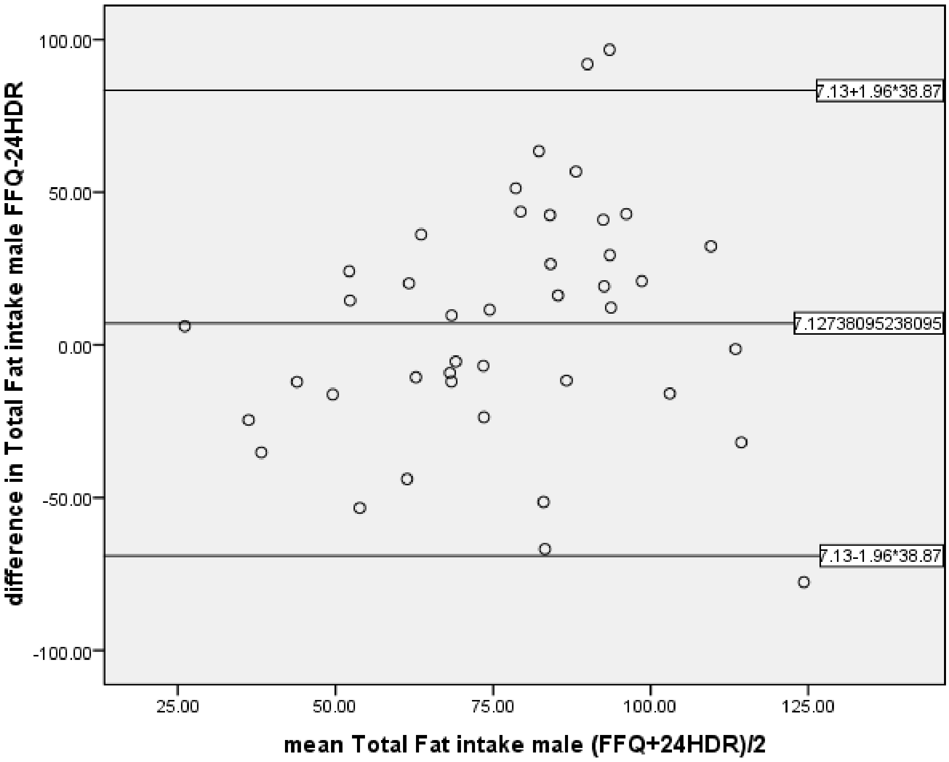

Plot for Saturated Fat intake (male)

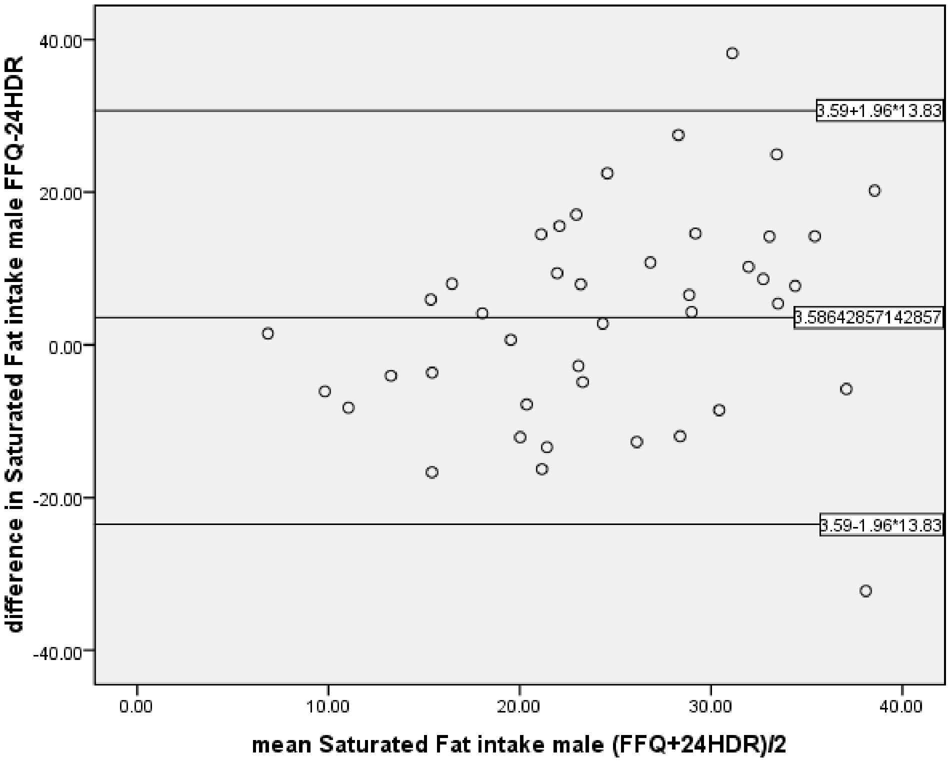

Plot for Monounsaturated Fat intake (male)

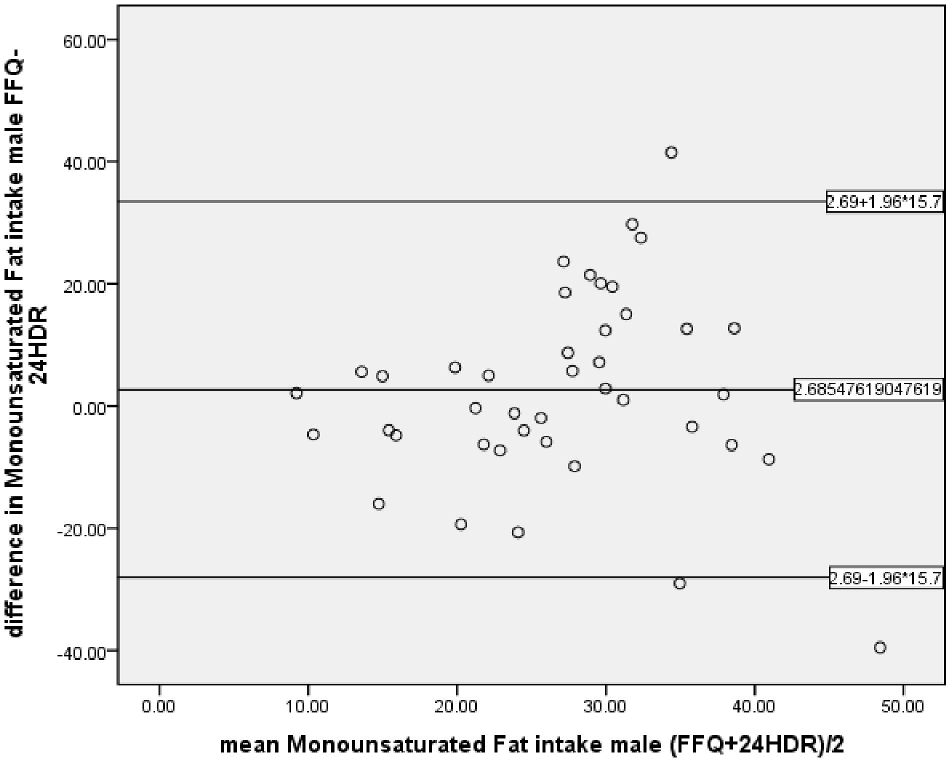

Plot for Polyunsaturated Fat intake (male)

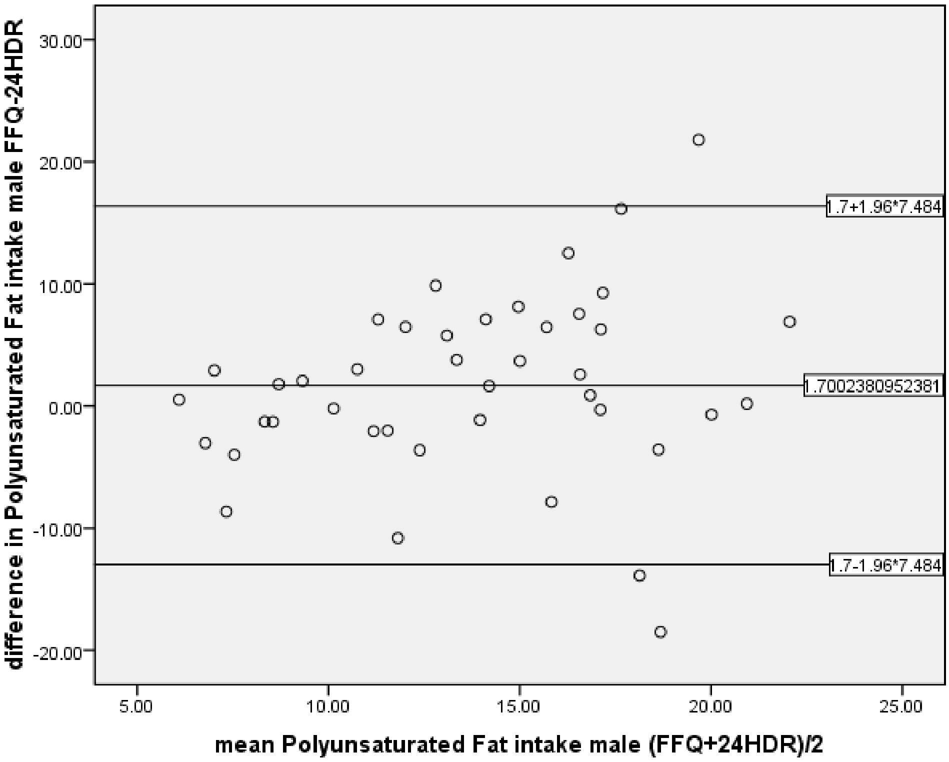

Plot for Cholesterol intake (male)

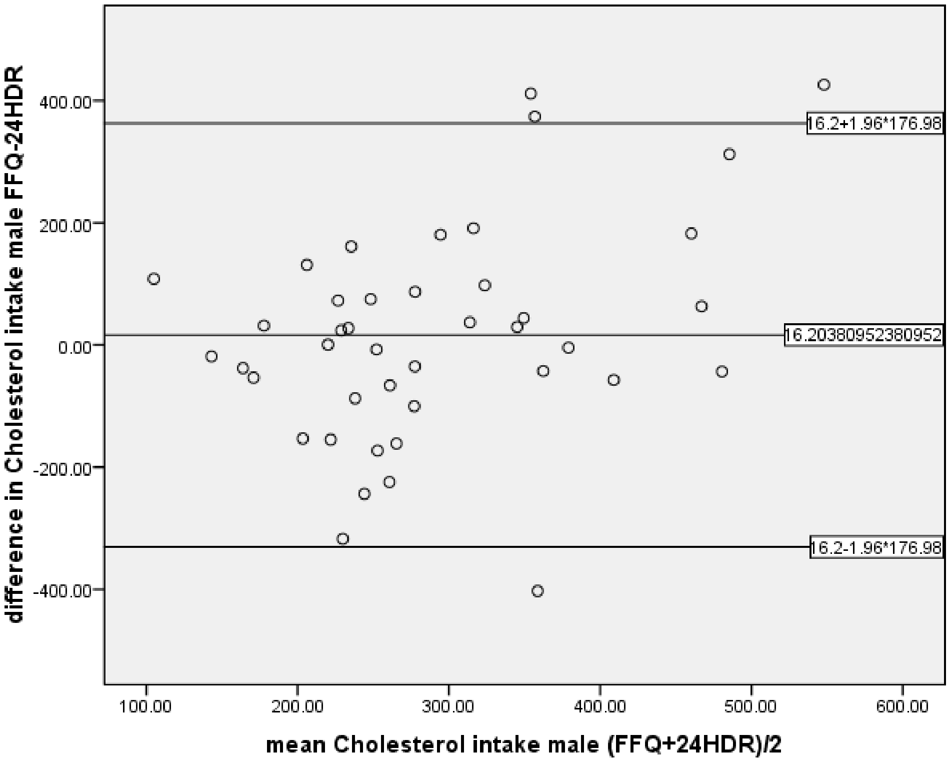

Plot for Vitamin A intake (male)

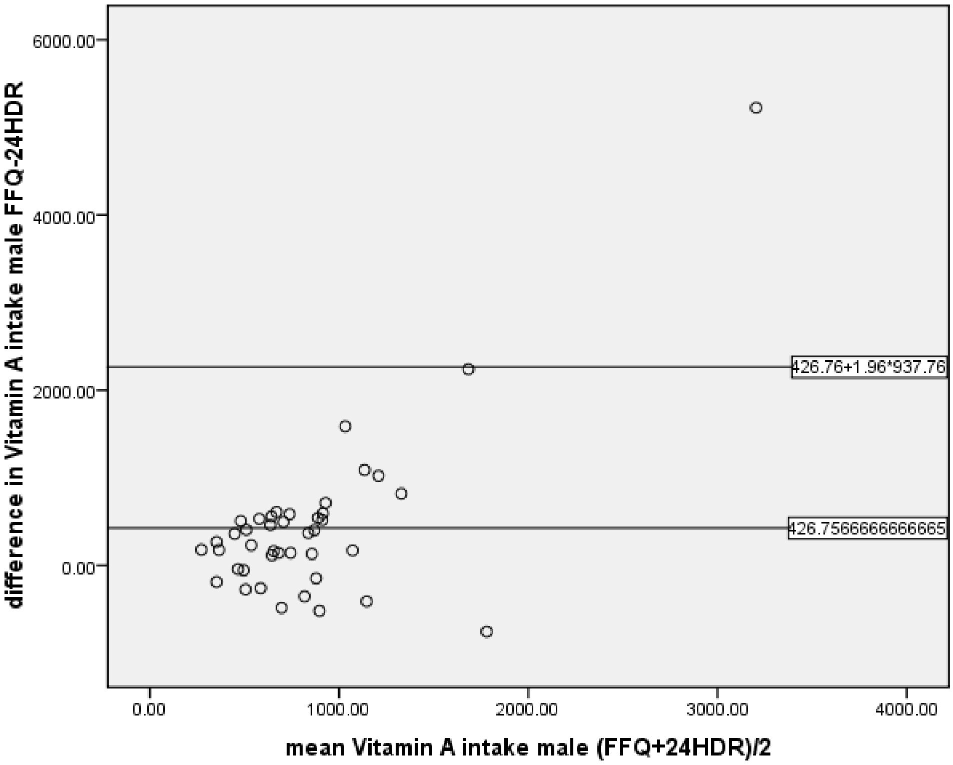

Plot for Carotene intake (male)

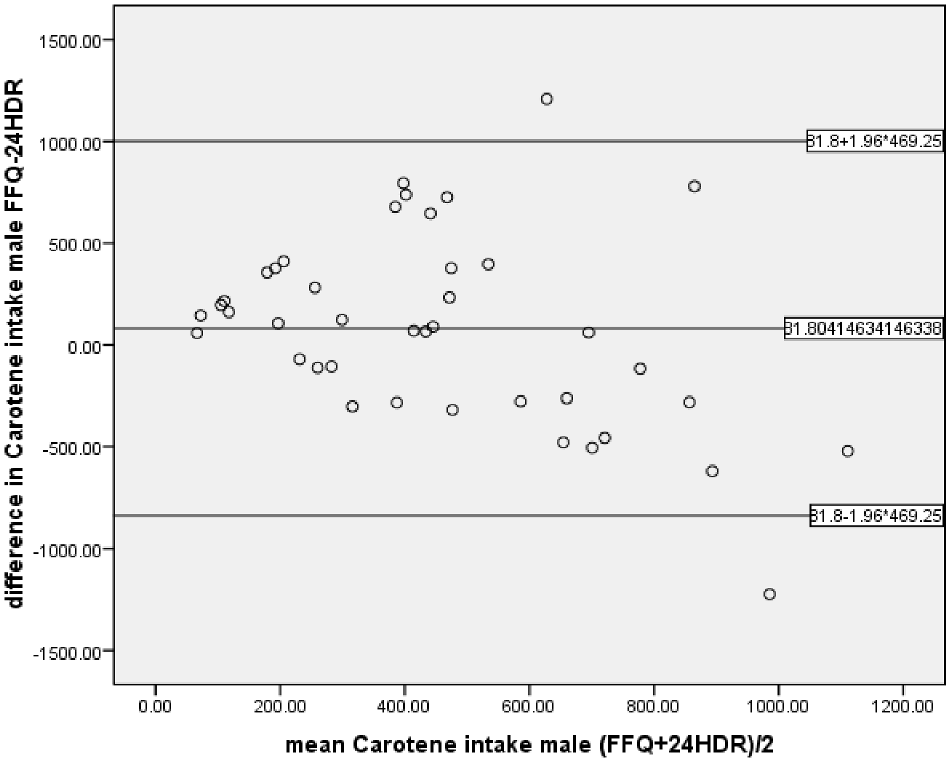

Plot for Vitamin D intake (male)

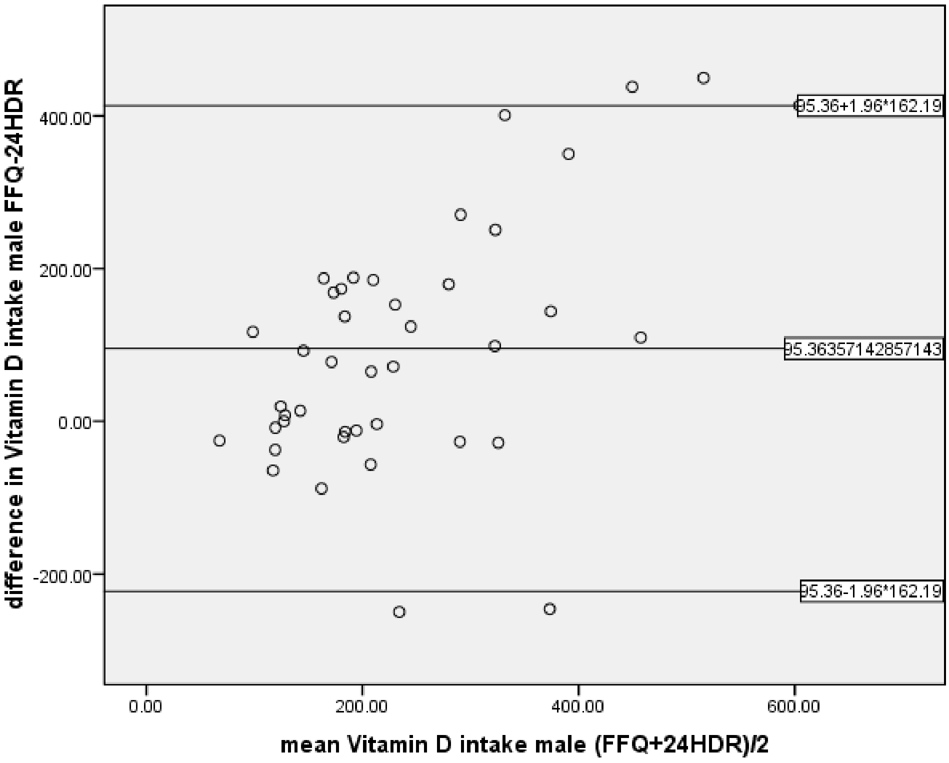

Plot for Calcium intake (male)

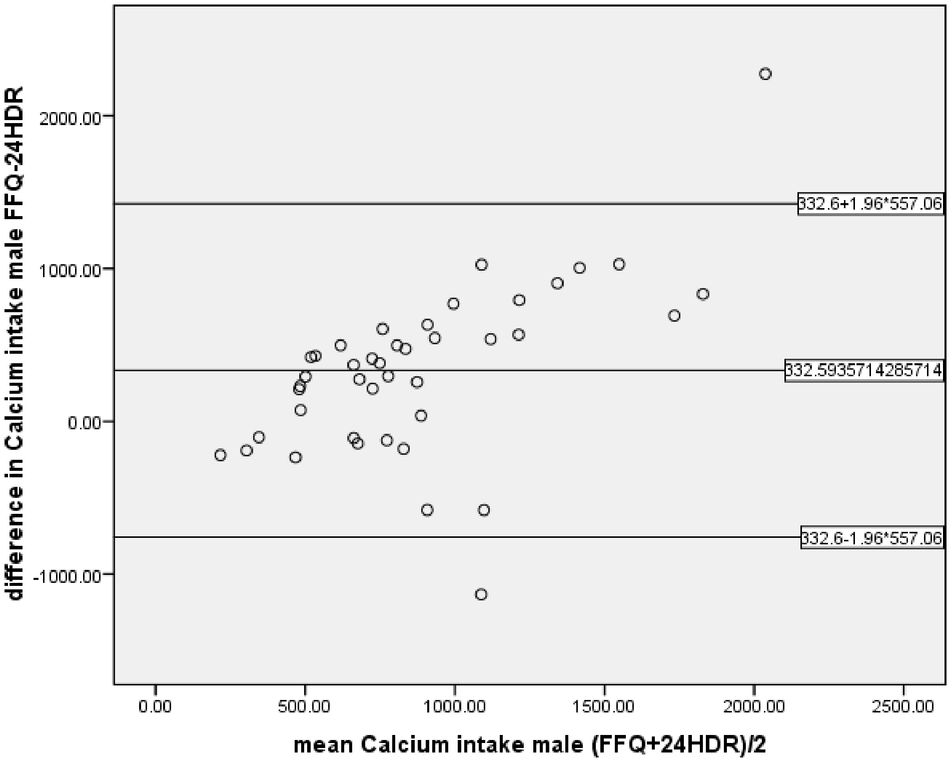

Supplement: Additional file 2 — Bland–Altman plots. [file 1475-2891-12-49-S2.pdf]
